# What do we know about frailty in the acute care setting? A scoping review

**DOI:** 10.1186/s12877-018-0823-2

**Published:** 2018-06-11

**Authors:** Olga Theou, Emma Squires, Kayla Mallery, Jacques S. Lee, Sherri Fay, Judah Goldstein, Joshua J. Armstrong, Kenneth Rockwood

**Affiliations:** 1 Department of Medicine, Dalhousie University, Camp Hill Veterans’ Memorial Building, 5955 Veterans’ Memorial Lane, Halifax, NS B3H 2E1 Canada; 2 Geriatric Medicine, QEII Health Sciences Centre, Nova Scotia Health Authority, Camp Hill Veterans’ Memorial Building, 5955 Veterans’ Memorial Lane, Halifax, NS B3H 2E1 Canada; 3 Sunnybrook Health Service, 2075 Bayview Avenue, BG-04, Toronto, ON M4N 3M5 Canada; 4 Emergency Health Services, 239 Brownlow Avenue, Suite 300, Dartmouth, NS B3B 2B2 Canada; 5 Department of Health Sciences, Lakehead University, 955 Oliver Road, Thunder Bay, ON P7B 5E1 Canada; 6 Division of Geriatric Medicine, Department of Medicine, Dalhousie University, Camp Hill Veterans’ Memorial Building, 5955 Veterans’ Memorial Lane, Halifax, NS B3H 2E1 Canada

**Keywords:** Frail elderly, Frailty, Aging, Acute care, Older adults, Scoping review

## Abstract

**Background:**

The ability of acute care providers to cope with the influx of frail older patients is increasingly stressed, and changes need to be made to improve care provided to older adults. Our purpose was to conduct a scoping review to map and synthesize the literature addressing frailty in the acute care setting in order to understand how to tackle this challenge. We also aimed to highlight the current gaps in frailty research.

**Methods:**

This scoping review included original research articles with acutely-ill Emergency Medical Services (EMS) or hospitalized older patients who were identified as frail by the authors. We searched Medline, CINAHL, Embase, PsycINFO, Eric, and Cochrane from January 2000 to September 2015.

**Results:**

Our database search initially resulted in 8658 articles and 617 were eligible. In 67% of the articles the authors identified their participants as frail but did not report on how they measured frailty. Among the 204 articles that did measure frailty, the most common disciplines were geriatrics (14%), emergency department (14%), and general medicine (11%). In total, 89 measures were used. This included 13 established tools, used in 51% of the articles, and 35 non-frailty tools, used in 24% of the articles. The most commonly used tools were the Clinical Frailty Scale, the Frailty Index, and the Frailty Phenotype (12% each). Most often (44%) researchers used frailty tools to predict adverse health outcomes. In 74% of the cases frailty predicted the outcome examined, typically mortality and length of stay.

**Conclusions:**

Most studies (83%) were conducted in non-geriatric disciplines and two thirds of the articles identified participants as frail without measuring frailty. There was great variability in tools used and more recently published studies were more likely to use established frailty tools. Overall, frailty appears to be a good predictor of adverse health outcomes. For frailty to be implemented in clinical practice frailty tools should help formulate the care plan and improve shared decision making. How this will happen has yet to be determined.

**Electronic supplementary material:**

The online version of this article (10.1186/s12877-018-0823-2) contains supplementary material, which is available to authorized users.

## Background

Providing health care to an aging population presents both challenges and opportunities. Perhaps most important is how we understand and respond to frailty. The concept of frailty was introduced in the literature of geriatric medicine and gerontology almost two decades ago to better understand the heterogeneous health status of the older persons. Since then, this area of research has grown exponentially. Although contested, frailty is increasingly understood conceptually as the increased vulnerability to adverse outcomes among people of the same chronological age [1]. Frail individuals can be thought of as complex systems close to failure, vulnerable to further physiological and psychological stressors caused by both intrinsic and environmental factors. Adding one more stressor to such a system, even a stress as minor as one more drug, may lead to a cascade effect. Not all older adults are frail. Among community-dwelling people over the age of 50 years approximately 20% are frail [2, 3]. Even so, levels of frailty are higher among those seen in clinical settings [4].

Frail patients have higher rates of adverse outcomes, and thus require adaptations of care, personalization of interventions, and modifications of standard protocols [1, 5]. As such, identifying frailty early in clinical care is vital [6–8]. Given its pervasive impact on health and the outcomes of health care, it has been proposed that frailty be considered routinely when treating the older patient [6]. To do so we need validated tools with sound measurement properties. With various instruments having been developed, there is heated debate over how to best operationally define frailty and which tools can feasibly be used across care settings [9, 10]. A 2007 systematic review identified 27 articles that included a frailty measure in older adults [11]. Since then many more tools have been developed and multiple systematic reviews have been published on them mostly focusing on community settings [2, 9, 12–15]. In a systematic review by de Vries and colleagues in 2011, 20 frailty tools were identified and the authors concluded that at that point the Frailty Index seemed to be the most suitable instrument to measure frailty [9]. A year later Pailoux and colleagues found that 10 instruments have been used for screening for frailty in primary health care [13]. Choosing among the many options available can be confusing for health care professionals.

Older frail adults are more vulnerable to health crises. They are more likely to be hospitalized or to need critical care, use emergency medical services, and have a longer in-hospital length of stay [16, 17]. Many critical, life altering decisions are made in the acute care setting during these crises. There are indications that frailty identification and management will improve clinical decision making and health outcomes within acute care. For example, frailty assessment by EMS and ED providers could facilitate referral or transport to the most appropriate service and could assist with identifying patients who will not benefit from aggressive medical treatments. Even so, before translational research programs focus on how frailty measures will be incorporated into every day care in the acute care setting and how they will benefit clinical decisions, we need to agree on how frailty will be measured and managed in this setting. Currently, no systematic reviews have been conducted with a general focus on EMS and in-hospital settings. Given the growing numbers of frail patients and the greater use of frailty tools within clinical settings, we conducted a scoping review to map and synthesize the literature around frailty in the acute care setting. This included identifying and documenting the nature and extent of research evidence related to frailty measurement and management in EMS and in-hospital settings.

## Methods

### Search strategy and selection criteria

This scoping review included original research articles published since January 2000. Two of the most commonly used frailty definitions, the Frailty Phenotype and the Frailty Index, were developed in 2001 which is when the literature around frailty started increasing. In order for the articles to be included, the following criteria had to be met: at least 50% of the participants were acutely-ill EMS (paramedic services) or hospitalized patients, at least one patient was aged 65 or older, and at least one participant was identified as frail by the authors (with or without reporting on measuring frailty). We did not limit articles by language or study design. Articles were excluded if they focused on care delivery outside of hospital or on hospitalized patients who were not acutely ill (for example, dialysis and chemotherapy outpatients, inpatients on rehabilitation wards or doing elective surgeries, and subacute patients).

We searched a wide range of academic literature databases including Medline, CINAHL, Embase, PsycINFO, Eric, and Cochrane up to September 2015. The search terms we used were all words that are used interchangeably as descriptors of “frailty”, “aging”, “pre-hospital”, and “acute care” in Medical Subject Headings, text, and keywords (Additional file 1: Table S1). Additional articles were identified by manually searching the reference lists of systematic reviews focusing on frailty. We contacted authors when we needed additional information about the eligibility of an article. Two members of the review team independently screened the title and abstracts and then the full text of the articles that met the inclusion criteria. Disagreements between the two reviewers were resolved by a third reviewer. We included reviewers who were fluent in all relevant languages.

### Data analysis

The database search results were uploaded into Refworks where duplicates were removed. DistillerSR software was used to manage the screening process. An excel data extraction form was developed to guide collection of information relevant to the review. The following descriptive data was extracted from each article that satisfied the inclusion criteria: year of publication, language, and country. For the articles that included a frailty measure we extracted additional descriptive data: number of participants, participant age, participant sex, study setting (medical discipline) and design, when and how frailty was measured, who completed the evaluation, the stated purpose for measuring frailty, the prevalence of frailty, and any adverse outcome measure examined in association with frailty. Analyses were conducted using IBM SPSS 21.

## Results

**Fig. 1:**
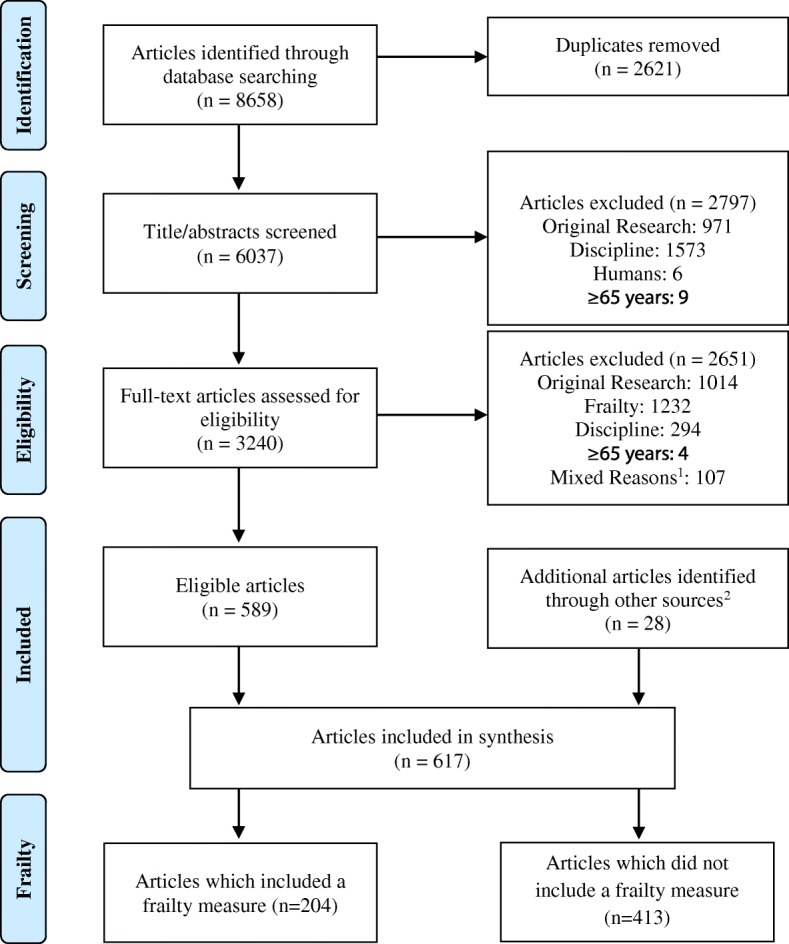
Flow chart. ^1^The two reviewers agreed that these articles should be excluded but disagreed on the reason for exclusion. ^2^Additional articles were identified by manually searching the reference lists of systematic reviews focusing on frailty

The initial database search resulted in 8658 articles. After duplicates were removed (*n* = 2621), we screened the title and abstract of 6037 articles and excluded 2797 from additional screening. Full text was obtained for 3240 articles, 589 of which remained after screening. We also hand-searched the reference list of other relevant papers, including systematic reviews focusing on frailty; 28 additional articles were added to bring the final number of included articles to 617 (Fig. 1; Additional file 1: Table S2).

In 67% of the articles (*n* = 413/617) the authors identified their participants as frail but did not provide an operational definition of frailty or refer to use of a frailty measurement tool (Fig. 1). Table 1 shows the descriptive characteristics of the articles that measured frailty (33%, *n* = 204) [18–221], whereas Additional file 1: Table S3 shows the descriptive characteristics of the articles that did not include a frailty measurement.

**Table 1:** Descriptive characteristics of the articles which included a frailty measure

	All	Geriatrics	Emergency Department	General Medicine	Cardiology	Orthopedics	Intensive Care Unit	Oncology	Surgery	Prehospital	General Medicine & Surgery	General Medicine & Geriatrics	Geriatrics & Oncology
Articles n (%)	204 (100)	29 (14.2)	29 (14.2)	22 (10.8)	20 (9.8)	11 (5.4)	9 (4.4)	6 (2.9)	5 (2.5)	2 (1)	18 (8.8)	7 (3.4)	2 (1)
Year n (%)													
2011–2015	141 (69.1)	14 (48.3)	25 (86.2)	19 (86.4)	16 (80.0)	11 (100.0)	9 (100)	3 (50.0)	5 (100.0)	2 (100.0)	6 (33.3)	2 (28.6)	1 (50.0)
2006–2010	39 (19.1)	10 (34.5)	3 (10.3)	3 (13.6)	2 (10.0)	0	0	2 (33.3)	0	0	5 (27.8)	2 (28.6)	0
2000–2005	24 (11.8)	5 (17.2)	1 (3.4)	0	2 (10.0)	0	0	1 (16.7)	0	0	7 (38.9)	3 (42.9)	1 (50.0)
Language n (%)													
English	200 (98.0)	29 (100.0)	27 (93.1)	21 (95.5)	20 (100.0)	11 (100.0)	9 (100.0)	6 (100.0)	4 (80.0)	2 (100.0)	18 (100.0)	7 (100.0)	2 (100.0)
Italian	2 (1.0)	0	1 (3.4)	0	0	0	0	0	1 (20.0)	0	0	0	0
Portuguese	2 (1.0)	0	1 (3.4)	1 (4.5)	0	0	0	0	0	0	0	0	0
Country n (%)													
United States	55 (27.0)	3 (10.3)	11 (37.9)	1 (4.5)	6 (30.0)	3 (27.3)	2 (22.2)	1 (16.7)	3 (60.0)	0	15 (83.3)	0	0
United Kingdom	23 (11.3)	4 (13.8)	3 (10.3)	5 (22.7)	2 (10.0)	2 (18.2)	1 (11.1)	0	0	0	1 (5.6)	0	1 (50.0)
Canada	18 (8.8)	5 (17.2)	2 (6.9)	2 (9.1)	1 (5.0)	1 (9.1)	4 (44.4)	0	0	1 (50.0)	0	0	0
Australia/New Zealand	16 (7.8)	1 (3.4)	1 (3.4)	2 (9.1)	1 (5.0)	1 (9.1)	1 (11.1)	0	0	0	1 (5.6)	0	1 (50.0)
Italy	14 (6.9)	3 (10.3)	2 (6.9)	3 (13.6)	0	0	0	4 (66.7)	1 (20.0)	0	0	1 (14.3)	0
Belgium	11 (5.4)	6 (20.7)	1 (3.4)	2 (9.1)	1 (5.0)	1 (9.1)	0	0	0	0	0	0	0
Spain	8 (3.9)	2 (6.9)	2 (6.9)	0	3 (15.0)	0	0	0	0	0	0	0	0
Denmark	8 (3.9)	0	2 (6.9)	2 (9.1)	0	3 (27.3)	0	0	0	0	0	1 (14.3)	0
Netherlands	8 (3.9)	1 (3.4)	0	0	0	0	0	0	0	0	1 (5.6)	0	0
Sweden	6 (2.9)	0	2 (6.9)	0	0	0	0	0	0	1 (50.0)	0	0	0
Norway	5 (2.5)	0	0	1 (4.5)	0	0	0	0	0	0	0	4 (57.1)	0
Other Europe^a^	15 (7.4)	3 (10.3)	1 (3.4)	1 (4.5)	2 (10.0)	0	1 (11.1)	1 (16.7)	1 (20.0)	0	0	0	0
Other^b^	17 (8.3)	1 (3.4)	2 (6.9)	3 (13.6)	4 (20.0)	0	0	0	0	0	0	1 (14.3)	0
Number of participants													
# of articles reporting	201	28	29	21	19	11	9	6	5	2	18	7	2
Range	9–971,434	44–20,933	40–7532	34–3479	24–111,023	16–36,900	22–47,427	54–350	36–35,344	88–203	99–129,400	156–1568	37–111
Median (IQR)	206 (100.5–460)	164 (99.3–490.3)	161 (99.5–278)	254 (118.5–562)	309 (135–773)	145 (47–284)	421 (119–982)	103 (69.8–180.5)	41 (38.5–17,764)	N/A	259.5 (397–1435.3)	254 (254–254)	N/A
Mean age of participants													
# of articles reporting	155	24	21	18	17	9	9	3	2	1	6	7	1
Range	47.1–91.6	66.4–86.7	75–86.8	65–86.5	53.2–91.6	47.1–87)	60–84	62–73.1	72–76.9	82.2	60.4–77.3	60.8–82	74.3
Median (IQR)	78.9 (74–82.9)	82.9 (79.9–84.1)	78 (76.8–81.3)	82.2 (77.9–84.1)	76 (69.9–80.3)	83.9 (77.3–86.3)	76.9 (70–80.8)	70.8 (N/A)	N/A	N/A	74.1 (68.3–75.1)	81.8 (72.8–81.8)	N/A
Percentage of females													
# of articles reporting	187	27	27	20	19	11	9	6	4	1	15	7	1
Range	2–100	2.5–81.7	28–83	21.6–67.2	16.3–60	4.3–83	38.7–69.7	40.1–100	22.5–54.4	62.1	2–57	53–74.8	46
Median (IQR)	54 (43.5–63)	63.7 (56–70)	53 (41.7–56.4)	60.4 (53.6–62.5)	46 (39.3–50.7)	75.4 (36.8–77.7)	45 (42.5–53.1)	45.5 (40.6–66.1)	41.9 (24.5–54.1)	N/A	2.8 (2.1–43.5)	65 (54.6–65)	N/A

We only stratified by disciplines that have been included in at least 2 articles

*N/A* not applicable, # number, *IQR* interquartile range

^a^Austria; Finland; France; Germany; Ireland; Poland; Slovenia; Switzerland

^b^Brazil; China; India; Israel; Japan; Mexico; Taiwan; Multiple Countries

Most of the articles (*n* = 442/617) reported on studies conducted in a single medical discipline. The most common single disciplines were geriatrics, emergency department, general medicine, cardiology, and orthopedics. A total of 49 studies were conducted in two disciplines, with general medicine and surgery (*n* = 25) being the most common combination followed by geriatric medicine and general medicine (*n* = 15). In 29 articles, the authors specified that participants were recruited from at least 3 disciplines and in 97 articles the authors did not specify from which discipline the hospitalized patients were recruited (Additional file 1: Figure S1).

The number of articles increased over time with more articles published in the last 5 years of our review; 52% (*n* = 318/617) were published between 2011 and 2015, with less than 20% (*n* = 121) published in 2005 or earlier. The increase in publications was even more striking among articles which measured frailty: 69% (*n* = 141/204) of articles published in 2011–2015, up from only 12% (*n* = 24) of those published in 2000–2005 (Table 1; Additional file 1: Figure S2A). Almost all included articles were written in English (94%, *n* = 583/617). The remaining articles were written in French (*n* = 11), Italian (*n* = 7) [93, 102], Spanish (*n* = 7), Dutch (*n* = 4), Portuguese (*n* = 3) [51, 141], and German (*n* = 2). Among the 34 non-English articles, only 4 measured frailty. A variety of countries were represented, with the majority, 59%, of the published studies conducted in Europe (*n* = 361/617), 25% in North America (*n* = 157), and 8% in Australia or New Zealand (*n* = 48). Among the 204 articles that measured frailty, 48% reported on studies conducted in Europe (*n* = 98) and 36% on studies conducted in North America (*n* = 73) (Table 1).

**Fig. 2:**
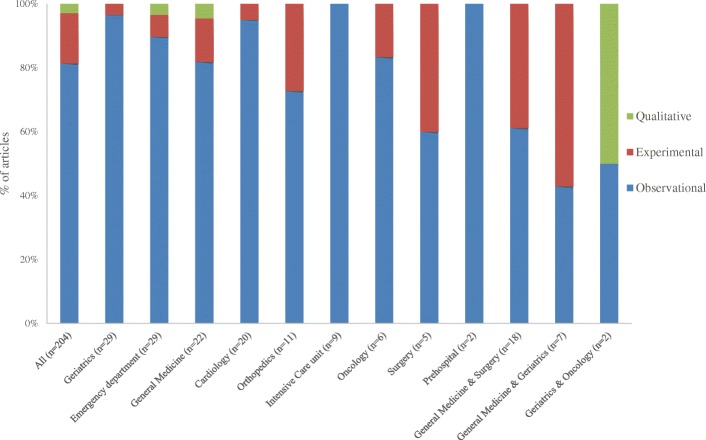
Proportion of articles that measured frailty by study design. We only stratified by disciplines that have been included in at least 2 articles

Based on the descriptive characteristic data extracted for the 204 articles that measured frailty, the number of participants per study ranged from 9 to 971,434 with a median of 206 participants (interquartile range 100.5–460). The mean age of participants ranged from 47.1 to 91.6 years with a median of 78.9 years (interquartile range 74–82.9); for 9 articles [26, 60, 111, 128, 136–138, 169, 211], the mean age was less than 65 years. Among the articles that reported the number of female and male participants (*n* = 187/204), the median was 54% females (interquartile range 43.5–63%) (Table 1). The majority of articles were observational studies (*n* = 166/204) and 16% were experimental (*n* = 32; 26 were randomized controlled trials). In total, 3% of the studies were qualitative (*n* = 6) [27, 69, 88, 124, 145, 181]; 3 studies examined the involvement of hospitalized older people in decision making regarding their care (Fig. 2).

Table 2 summarizes the characteristics of frailty measurement as reported in the included articles. In most cases, frailty was measured by either a health care professional (*n* = 80) or a researcher (*n* = 81) (Additional file 1: Figure S3). In geriatrics, oncology, surgery, and EMS disciplines, frailty assessments were done mostly by health care professionals, whereas in emergency department, general medicine, ICU, orthopedics, and cardiology it was more common that assessments were done by researchers (Additional file 1: Figure S3). Frailty was measured at hospital admission in almost half of the articles (*n* = 94), at discharge in 5% of the articles (*n* = 10), and was operationalized retrospectively through chart reviews or databases in 13% of the articles (*n* = 27). In cardiology, orthopedics, and EMS disciplines, more than one quarter of the articles operationalized frailty retrospectively (Table 2).

**Table 2:** Frailty charactseristics of included articles

	All	Geriatrics	Emergency Department	General Medicine	Cardiology	Orthopedics	Intensive Care Unit	Oncology	Surgery	Prehospital	General Medicine & Surgery	General Medicine & Geriatrics	Geriatrics & Oncology
Articles n (%)	240 (100)	29 (12.1)	29 (12.1)	22 (9.2)	20 (8.3)	11 (4.6)	9 (3.8)	6 (3)	5 (2.1)	2 (0.8)	18 (7.5)	7 (2.9)	2 (0.8)
When was frailty measured n (%)													
At admission	94 (46.1)	15 (51.7)	18 (62.1)	12 (54.5)	6 (30.0)	3 (27.3)	5 (55.6)	3 (50.0)	3 (60.0)	0	3 (16.7)	6 (85.7)	1 (50.0)
During hospitalization	34 (16.7)	9 (31.0)	3 (10.3)	5 (22.7)	4 (20.0)	2 (18.2)	1 (11.1)	2 (33.3)	0	1 (50.0)	3 (16.7)	0	0
At discharge	10 (4.9)	1 (3.4)	1 (3.4)	0	3 (15.0)	0	1 (11.1)	0	0	0	0	0	0
Mixed^a^	13 (6.4)	1 (3.4)	3 (10.3)	2 (9.0)	0	1 (9.1)	0	1 (16.7)	0	0	0	1 (14.3)	0
Chart/Database review	27 (13.2)	3 (10.3)	3 (10.3)	2 (9.1)	6 (30.0)	3 (27.3)	2 (22.2)	0	1 (20.0)	1 (50.0)	1 (5.6)	0	0
Not reported	26 (12.7)	0	1 (3.4)	1 (4.5)	1 (5.0)	2 (18.2)	0	0	1 (20.0)	0	11 (61.1)	0	1 (50.0)
Number of frailty measures used n (%)													
1	185 (90.7)	25 (86.2)	27 (93.1)	21 (95.5)	16 (80.0)	8 (72.7)	8 (88.9)	6 (100.0)	4 (80.0)	2 (100.0)	18 (100.0)	7 (100.0)	2 (100.0)
2	9 (4.4)	3 (10.3)	0	0	4 (20.0)	0	1 (11.1)	0	0	0	0	0	0
3	5 (2.5)	0	2 (6.9)	1 (4.5)	0	2 (18.2)	0	0	0	0	0	0	0
4	4 (2.0)	1 (3.4)	0	0	0	1 (9.1)	0	0	0	0	0	0	0
6	1 (0.5)	0	0	0	0	0	0	0	1 (20.0)	0	0	0	0
Type of frailty measure n (%)													
Established frailty tools	123 (51.3)	17 (48.6)	17 (51.5)	17 (70.8)	16 (66.7)	6 (33.3)	9 (90.0)	3 (50.0)	6 (60.0)	1 (50.0)	0	6 (85.7)	1 (50.0)
Non-frailty tools^b^	57 (23.8)	11 (31.4)	13 (39.4)	4 (16.7)	3 (12.5)	10 (55.6)	0	1 (16.7)	4 (40.0)	0	1 (5.6)	0	0
Ad hoc measures^c^	56 (23.3)	6 (17.1)	3 (9.1)	3 (12.5)	4 (16.7)	2 (11.1)	1 (10.0)	2 (33.3)	0	1 (50.0)	15 (83.3)	1 (14.3)	1 (50.0)
Clinical judgment	4 (1.7)	1 (2.9)	0	0	1 (4.2)	0	0	0	0	0	2 (11.1)	0	0
Frailty Measure used n (%)													
*Established frailty tools*													
Clinical Frailty Scale	28 (11.7)	6 (17.1)	4 (12.1)	4 (16.7)	3 (12.5)	0	6 (60.0)	0	0	0	0	0	0
Frailty Phenotype	28 (11.7)	2 (5.7)	4 (12.1)	4 (16.7)	6 (25.0)	1 (5.6)	2 (20.0)	0	2 (20.0)	0	0	1 (14.3)	0
Frailty Index	28 (11.7)	3 (8.6)	7 (21.2)	4 (16.7)	1 (4.2)	4 (22.2)	1 (10.0)	0	1 (10.0)	1 (50.0)	0	1 (14.3)	0
Edmonton Frailty Scale	10 (4.2)	0	2 (6.1)	2 (8.3)	2 (8.3)	1 (5.6)	0	0	0	0	0	0	0
Winodgrad Index	7 (2.9)	0	0	1 (4.2	0	0	0	0	0	0	0	4 (57.1)	0
Balducci criteria	5 (2.1)	0	0	0	0	0	0	3 (50.0)	1 (10.0)	0	0	0	1 (50.0)
Rockwood Geriatric Frailty Status	5 (2.1)	2 (5.7)	0	0	1 (4.2)	0	0	0	1 (10.0)	0	0	0	0
Study of Osteoporotic Fracture Index	3 (1.3)	2 (5.7)	0	1 (4.2)	0	0	0	0	0	0	0	0	0
Tilburg Frailty Indicator	3 (1.3)	0	0	1 (4.2)	2 (8.3)	0	0	0	0	0	0	0	0
FRAIL scale	2 (0.8)	1 (2.9)	0	0	1 (4.2)	0	0	0	0	0	0	0	0
Groningen Frailty Indicator	2 (0.8)	0	0	0	0	0	0	0	1 (10.0)	0	0	0	0
Other established tools^d^	2 (0.8)	1 (2.9)	0	0	0	0	0	0	0	0	0	0	0
*Non-frailty tools*^b^													
Identification of Seniors at Risk	12 (5.0)	4 (11.4)	3 (9.1)	2 (8.3)	1 (4.2)	1 (5.6)	0	0	0	0	0	0	0
Vulnerable Elders Survey	5 (2.1)	0	3 (9.1)	0	0	0	0	0	1 (10.0)	0	0	0	0
Grip strength	4 (1.7)	2 (5.7)	0	0	0	1 (5.6)	0	0	0	0	0	0	0
Barthel Index	2 (0.8)	0	2 (6.1)	0	0	0	0	0	0	0	0	0	0
Life Space Assessment	2 (0.8)	0	2 (6.1)	0	0	0	0	0	0	0	0	0	0
Multidimensional Prognosis Index	2 (0.8)	2 (5.7)	0	0	0	0	0	0	0	0	0	0	0
Short Physical Performance Battery	2 (0.8)	1 (2.9)	0	0	0	0	0	1 (16.7)	0	0	0	0	0
Other non-frailty tools^e^	28 (11.7)	2 (5.7)	3 (9.1)	2 (9.1)	2 (8.3)	8 (44.4)	0	0	3 (30.0)	0	1 (5.6)	0	0
*Ad hoc measures*^c^													
GEM criteria^f^	12 (5.0)	0	0	0	0	0	0	0	0	0	12 (66.7)	0	0
Other ad hoc measures	44 (18.3)	6 (17.1)	3 (9.1)	3 (12.5)	4 (16.7)	2 (11.1)	1 (10.0)	2 (33.3)	0	1 (50.0)	3 (16.7)	1 (14.3)	1 (50.0)
*Clinical judgment*	4 (1.7)	1 (2.9)	0	0	1 (4.2)	0	0	0	0	0	2 (11.1)	0	0
Purpose of frailty use n (%)													
Risk stratification^g^	89 (43.6)	12 (41.4)	15 (51.7)	10 (45.5)	11 (55.0)	8 (72.7)	8 (88.9)	2 (33.3)	2 (40.0)	1 (50.0)	3 (16.7)	2 (28.6)	0
Inclusion/Exclusion criterion	41 (20.1)	4 (13.8)	3 (10.3)	4 (18.2)	1 (5.0)	0	0	0	1 (20.0)	1 (50.0)	13 (72.2)	5 (71.4)	0
Outcome measure	4 (2.0)	1 (3.4)	1 (3.4)	0	1 (5.0)	1 (9.1)	0	0	0	0	0	0	0
Mixed^h^	3 (1.5)	0	0	2 (9.0)	0	0	0	1 (16.7)	0	0	0	0	0
Only descriptive^i^	67 (32.8)	12 (41.4)	10 (34.5)	6 (27.3)	7 (35.0)	2 (18.2)	1 (11.1)	3 (50.0)	2 (40.0)	0	2 (11.1)	0	2 (100.0)
Prevalence of frailty (%)													
# of articles reporting	122	17	13	12	17	5	8	5	7	0	4	2	1
Range	1.1–100	1.1–90.0	6.9–78.0	23.0–77.9	4.7–77.8	11.4–89.4	13.7–100	19.1–96.7	29.3–79.9	N/A	30.3–91.0	41.4–59.0	26.0
Median (IQR)	48.5 (32.0–69.5)	43.3 (26.9–70.7)	46.7 (34.2–70.2)	51.3 (34.8–55.2)	35.5 (28.7–69)	68.5 (31.2–89.4)	33.5 (25.3–47.0	41.4 (26.7–81.0)	54.3 (50.0–70.7)	N/A	45.3 (32.2–81.4)	N/A	N/A
Adverse outcomes predicted by frailty [n predicted/total measured (% predictive)]^j^													
*All adverse outcomes*	169/228 (74.1)	23/30 (69.7)	25/32 (78.1)	18/22 (81.8)	15/19 (78.9)	11/16 (68.8)	22/33 (66.7)	4/8 (50)	11/14 (78.6)	1/1 (100)	4/4 (100)	2/3 (66.7)	0
*Mortality*	58/69 (84.1)	13/14 (92.9)	6/7 (85.7)	4/4 (100.0)	7/10 (70.0)	4/5 (80.0)	6/8 (75.0)	1/2 (50.0)	6/7 (85.7)	1/1 (100.0)	0	0	0
*Length of stay*	24/33 (72.7)	3/5 (60.0)	4/5 (80.0)	4/7 (57.1)	2/2 (100.0)	3/3 (100.0)	3/6 (50.0)	0	0	0	1/1 (100.0)	2/2 (100.0)	0
*In-hospital complications*	20/29 (69.0)	0	2/2 (100.0)	0	2/2 (100.0)	1/4 (25.0)	1/3 (33.3)	1/2 (50.0)	5/7 (71.4)	0	1/1 (100.0)	0	0
*Institutionalization*	27/29 (93.1)	1/1 (100.0)	6/6 (100.0)	7/7 (100.0)	1/1 (100.0)	1/1 (100.0)	5/7 (71.4)	1/1 (100.0)	0	0	0	0	0
*Rehospitalization*	9/18 (50.0)	3/4 (75.0)	3/5 (60.0)	0/1 (0)	1/1 (100.0)	0	0/1 (0)	0	0	0	1/1 (100.0)	0	0
*Functional decline*	8/8 (66.7)	2/2 (100.0)	2/4 (50.0)	1/1 (100.0)	0	0	3/3 (100.0)	0	0	0	0	0/1 (0)	0
*Treatment change*^k^	8/8 (66.7)	1/1 (100.0)	0/1 (0)	0	2/3 (66.7)	0	2/3 (66.7)	0/1 (0)	0	0	1/1 (100.0)	0	0
*Falls*	3/7 (42.9)	0/4 (0)	1/1 (100.0)	0	0	0	0	0	0	0	0	0	0
*Delirium*	1/4 (25.0)	0/2 (0)	0	1/1 (100.0)	0	0/1 (0)	0	0	0	0	0	0	0
*Treatment response*^l^	4/4 (100.0)	0	0	1/1 (100.0)	0	0	0	1/1 (100.0)	0	0	0	0	0
*Cognitive decline*	0/3 (0)	0	0	0	0	0	0	0	0	0	0	0	0
*Quality of life*	2/2 (100.0)	0	0	0	0	1/1 (100.0)	1/1 (100.0)	0	0	0	0	0	0
*Other*^m^	5/6 (83.3)	0	1/2 (50.0)	0	0	1/1 (100.0)	1/1 (100.0)	0	0	0	0	0	0

We only stratified by disciplines that have been included in at least 2 articles

*N/A* not applicable, *#* number, *IQR* interquartile range

^a^Multiple testings for each patient or different testing times for subgroup of patients

^b^Tools that were not developed specifically for measuring frailty

^c^Operationalized definitions of frailty (for example, ≥65 years of age plus 3 comorbidities)

^d^Clinical Global Impression of Change in Physical Frailty; Geriatrician Clinical Impression of Frailty

^e^Non-frailty tools that were used only once

^f^The Geriatric Evaluation and Management (GEM) drug study criterion was meeting at least 2 of the following 10 criteria: dependence in at least one activity of daily living, stroke within 3 months, previous falls, difficulty ambulating, malnutrition, dementia, depression, unplanned admission in the last 3 months, prolonged bed rest, or incontinence

^g^Articles that examined the association of a frailty measure with a longitudinal adverse outcome

^h^Inclusion/Exclusions criterion and outcome measure; risk stratification and outcome measure

^i^Articles which only used frailty as a descriptive characteristic. For example, if an article used frailty both as an inclusion/exclusion criterion and a descriptive characteristic, it was only categorized as inclusion/exclusion criterion in our review

^j^More than one outcome can be examined within each article

^k^For example, change in anticoagulant use

^l^For example, antibody levels

^m^Adverse outcomes that were examined only once

Overall, 89 measures were used 240 times in the 204 included articles. Most of the articles included only one frailty measure (*n* = 185), while 9 articles included 2 measures [45, 54, 70, 112, 163, 172, 183, 196, 212] and 10 articles included 3 or more measures (Table 2). Thirteen established tools, developed to measure frailty, were used in 51% of the cases (*n* = 123). Thirty-five non-frailty tools were used in 24% of the cases (*n* = 57). These were validated scales but not developed to identify frailty (e.g. short physical performance battery). In 23% of the cases (*n* = 56), ad hoc measures were used which were operational definitions developed for the purpose of that study (e.g. everyone who was older than 65 and had 2 or more chronic diseases was considered frail). Four articles used clinical judgment to define frailty. Established frailty tools were the most common type of measure across all disciplines, except the orthopedic discipline where non-frailty scales were more common (*n* = 10/18 articles) (Table 2). When the results were stratified by year of publication, we found that ad hoc measures of frailty (*n* = 15/24; 63%) were most commonly used in the oldest articles (those published between 2000 and 2005), whereas use of established frailty tools (*n* = 101/174; 58%) was most common in the most recent articles (published between 2011 and 2015), (Additional file 1: Figure S4). In articles reporting on studies conducted in USA, ad hoc measures were the most common (*n* = 26/62; 42%). In contrast, in the rest of the countries established frailty tools were the most common, with Canada (*n* = 17/18; 94%) and Australia/New Zealand (*n* = 14/18; 78%) having the highest proportion.

The Clinical Frailty Scale, the Frailty Index, and the Frailty Phenotype were the most common tools used to measure frailty (*n* = 28 each) (Additional file 1: Figure S5). The Clinical Frailty Scale was the most popular measure used in geriatric (*n* = 6/35; 17%) [34, 66, 94, 110, 185, 212] and ICU disciplines (*n* = 6/10; 60%) [103, 176, 190, 192, 196, 211] (Table 3), in Canada (*n* = 12/18; 67%) and the UK (*n* = 8/26; 31%) [103, 116, 139, 180, 188, 202, 207, 213], and in observational articles (*n* = 26/202; 13%). The Frailty Index was the most popular measure used in emergency departments (*n* = 7/33; 21%) [93, 106, 125, 131, 163, 165, 195] and orthopedics (*n* = 4/18; 22%) [126, 189, 219, 220] (Table 3), in Italy (*n* = 5/19; 26%) [93, 104, 106, 133] and Denmark (*n* = 3/8; 38%) [189, 219, 220], and in experimental articles (*n* = 6/32; 19%) [50, 62, 166, 189, 219, 220]. The Frailty Phenotype was the most popular measure used in cardiology (*n* = 6/24; 25%) [45, 89, 90, 112, 172, 208] and surgery (*n* = 2/10; 20%) [184, 221] (Table 3), and in Spain (*n* = 4/10; 40%) [90, 112, 172, 174] and Sweden (*n* = 2/6, 33%) [92, 119]. Among the 122 articles that reported the prevalence of frailty, the median prevalence was 49%. The highest prevalence was in orthopedics (69% median frailty prevalence) and the lowest in ICU (34% median frailty prevalence) (Table 2).

**Table 3:** Ranking of the most commonly used measures by discipline

Ranked	All	Geriatrics (*n* = 29)	Emergency Department (*n* = 29)	General Medicine (*n* = 22)	Cardiology (*n* = 20)	Orthopedics (*n* = 11)	Intensive Care Unit (*n* = 9)	Oncology (*n* = 6)	Surgery (*n* = 5)	Prehospital (*n* = 2)	General Medicine & Surgery (*n* = 18)	General Medicine & Geriatrics (*n* = 7)	Geriatrics & Oncology (*n* = 2)
1	Clinical Frailty Scale (12%), Frailty Phenotype (12%), Frailty Index (12%)	Clinical Frailty Scale (17%)	Frailty Index (21%)	Clinical Frailty Scale (17%), Frailty Phenotype (17%), Frailty Index (17%)	Frailty Phenotype (25%)	Frailty Index (22%)	Clinical Frailty Scale (60%)	Balducci Criteria (50%)	Frailty Phenotype (20%)	Frailty Index (50%)	GEM Criteria^a^ (67%)	Winograd Index (57%)	Balducci Criteria (50%)
2		Identification of Seniors at Risk (11%)	Clinical Frailty Scale (12%), Frailty Phenotype (12%)		Clinical Frailty Scale (13%)	Frailty Phenotype (6%), Edmonton Frailty Scale (6%), Identification of Seniors at Risk (6%), Grip Strength (6%)	Frailty Phenotype (20%)	Short Physical Performance Battery (17%)	Frailty Index (10%), Groningen (10%), Balducci Criteria (10%), Rockwood Geriatric Frailty Status (10%), Vulnerable Elders Survey (10%)	N/A	N/A	Frailty Index (14%), Frailty Phenotype (14%)	N/A
3		Frailty Index (9%)			Edmonton Frailty Scale (8%), Tilburg Frailty Indicator (8%)		Frailty Index (10%)	N/A					
4	Identification of Seniors at Risk (5%), GEM Criteria^a^ (5%)	Frailty Phenotype (6%), Study of Osteoporotic Fracture Index (6%), Rockwood Geriatric Frailty Status (6%), Multidimensional Prognosis Index (6%), Grip Strength (6%)	Identification of Seniors at Risk (9%), Vulnerable Elders Survey (9%)	Edmonton Frailty Scale (8%), Identification of Seniors at Risk (8%)			N/A					N/A	
5					Frailty Index (4%), Rockwood Geriatric Frailty Status (4%), FRAIL scale (4%), Identification of Seniors At Risk (4%)								

We only stratified by disciplines that have been included in at least 2 articles

*N/A* not applicable

^a^The Geriatric Evaluation and Management (GEM) drug study criterion was meeting at least 2 of the following 10 criteria: dependence in at least one activity of daily living, stroke within 3 months, previous falls, difficulty ambulating, malnutrition, dementia, depression, unplanned admission in the last 3 months, prolonged bed rest, or incontinence

**Fig. 3:**
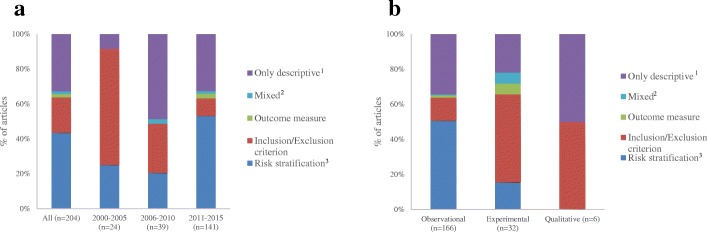
Proportion of articles based on the reason frailty measures were used in the articles. Stratified by (**a**). Year (**b**). Study design. ^1^Articles which only used frailty as a descriptive characteristic. For example, if an article used frailty both as an inclusion/exclusion criterion and a descriptive characteristic, it was only categorized as inclusion/exclusion criterion in our review. ^2^Inclusion/Exclusions criterion and outcome measure; risk stratification and outcome measure. ^3^Articles that examined the association of a frailty tool with a longitudinal adverse outcome

In almost half of the articles (*n* = 89, 44%), researchers used frailty for risk stratification purposes to examine whether frailty can predict adverse health outcomes (*n* = 89). In 41 articles (20%), frailty was used strictly as an inclusion criterion for patient recruitment. In 7 articles (4%), frailty was used solely as an outcome or in combination with risk stratification or inclusion criterion [52, 118, 119, 132, 138, 187, 216]. In the remaining articles (*n* = 67, 33%), frailty was only used as a descriptive of the sample (e.g. reporting only on the prevalence of frailty of the sample). Risk stratification was the most common reason for using the frailty tools across all disciplines, except in oncology where descriptive was the most common reason (Table 2). Stratified by year of publication (Fig. 3a), in the oldest articles (2000–2005) frailty tools were most commonly used as inclusion criterion (*n* = 16/24; 67%), while in the most recent articles (2011–2015) the most common use was risk stratification (*n* = 75/141; 53%); all 4 articles [118, 132, 138, 216] that used frailty solely as an outcome were published after 2010. In the included observational articles, risk stratification was the most common reason (*n* = 84/166; 51%) whereas in the experimental articles inclusion criterion was the most common (*n* = 16/32, 50%) (Fig. 3b). One of the experimental studies (prospective non-randomized trial) modified treatment plans based on frailty levels in cancer patients [52]. Three of the experimental studies (all randomized controlled trials) used frailty as an outcome measure [118, 119, 216]: two were published protocols (one of an exercise intervention and one of a combined exercise and nutrition intervention) and the other was a multi-professional team approach intervention creating a continuum of care for patients from the hospital emergency department to the older person’s own home (no significant change in frailty was found) [118, 216].

**Fig. 4:**
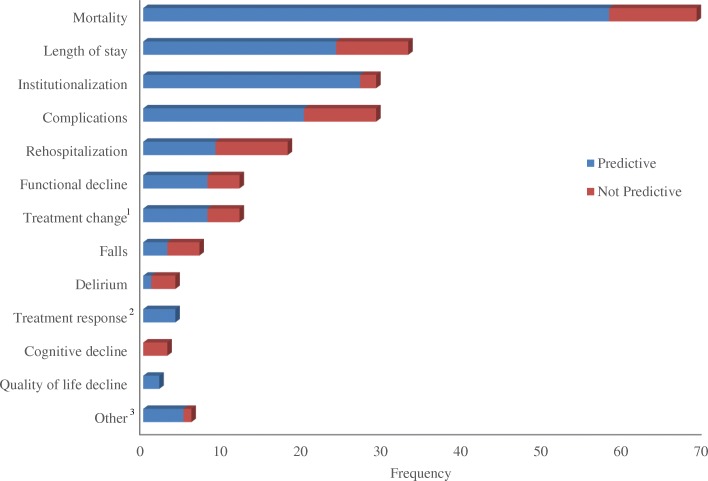
Number of times that the association of a frailty tool with a longitudinal outcome was examined. ^1^ For example, change in anticoagulant use. ^2^ For example, antibody levels. ^3^ Adverse outcomes that were examined only once

**Fig. 5:**
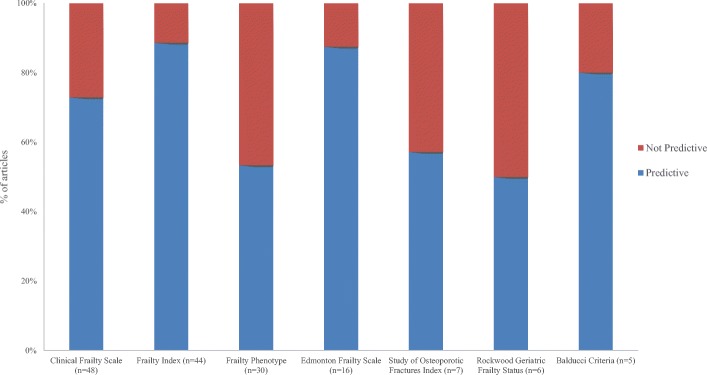
Proportion of articles demonstrating the association of frailty with longitudinal adverse outcomes stratified by frailty measure. We only presented the established frailty tools whose association with outcomes were examined at least 5 times

Among the 89 articles that used a frailty measure as a risk stratification tool, 115 measures were included; most often established frailty tools (*n* = 73, 64%), with the most common being the Frailty Index (*n* = 23, 20%), the CFS (*n* = 19, 17%), the Frailty Phenotype (*n* = 12, 10%), and the Edmonton frailty scale (*n* = 6, 5%) [71, 86, 121, 149, 178, 179]. Among these 89 risk stratification articles, 228 adverse outcomes were examined. The most frequent outcomes examined were mortality, in-hospital length of stay, institutionalization, and complications. Overall, in 169 cases (74%), frailty was predictive of an outcome (i.e. statistical significant association between frailty and the outcome measure), whereas in 59 cases it was not (26%). Frailty was predictive of mortality in 84% of the articles where this outcome was examined, in-hospital length of stay in 73%, institutionalization in 93%, and complications in 69% (Fig. 4). The highest rate for significant prediction of all outcomes combined was in EMS (*n* = 1/1, 100%) [159] and general medicine (*n* = 18/22, 82%) disciplines. The lowest rate was in oncology (*n* = 4/8, 50%) [49, 52, 169]. For mortality, the highest rate for significant prediction was in general medicine (*n* = 4/4, 100%) [99, 101, 116, 156] and the lowest in oncology (*n* = 1/2, 50%) [49] and cardiology (*n* = 7/10, 70%) [89, 121, 128, 172, 173, 208, 210]. When we stratified analysis by type of tool, the significant prediction rate was 74% (*n* = 122/164) for the established frailty tools, 84% for the ad-hoc tools (*n* = 16/19), 100% for clinical judgment (*n* = 2/2) [36, 111], and 67% for the non-frailty scales (*n* = 29/43). When we stratified by scale used, the predictive rate among the four most commonly used scales were 89% for the Frailty Index (*n* = 39/44 outcomes), 88% for the Edmonton frail scale (14/16 outcomes), 73% for the CFS (*n* = 35/48), and 53% for the Frailty Phenotype (*n* = 16/30) (Fig. 5).

## Discussion

We identified and documented the nature and extent of research evidence related to frailty measurement and management in the pre-hospital and EMS acute care setting. We found that most articles were done in non-geriatric disciplines between 2011 and 2015 in North America or Europe. Two thirds of the articles identified participants as frail without measuring frailty, and as time passed, more frailty studies were conducted across all disciplines. Overall, 89 measures were used including 13 established tools and 35 non-frailty tools; more recent studies were more likely to use established frailty tools. We found that the most commonly used scales were the Clinical Frailty Scale, the Frailty Index, and the Frailty Phenotype. Most articles were observational and used frailty tools to predict adverse health outcomes, especially mortality and institutionalization. Overall, frailty seems to be a good predictor of adverse health outcomes; in 74% of the cases frailty was predictive. When we looked at specific scales, the Frailty Index and the Edmonton scale seemed to have the best predictive ability.

This scoping review has a number of limitations. Quality assessments or meta-analysis were not conducted, and the scope of the review was very broad; for example, if participants were called frail once in the discussion the article was included. This was an appropriate first step for the field of frailty within acute care. Now that we have identified specific areas with sufficient research evidence, the next step for the field is to answer more focused research questions, such as a meta-analysis on the ability of the frailty scales to predict mortality in acute care. Another limitation of our scoping review is that non-English articles may have been missed because the databases searched included mostly English journals. Also, we only included articles with acutely-ill inpatients, which means we have not gathered data on other articles that focused on frailty, for example, those in rehabilitation and dialysis units, and elective surgery. Finally, the grey literature was not reviewed.

Our scoping review includes articles published up to September 2015. More frailty papers have since been published. Due to the broad scope of our review and the pace of frailty research, it is not feasible to conduct a completely up to date review. We are confident that the main findings and gaps identified continue to be relevant. For example, three systematic reviews published in 2017 examined frailty in surgical patients and showed similarly to our study that the evidence about the ability of frailty tools to predict adverse health outcomes in surgical patients is limited and that even less studies have tested whether interventions can improve perioperative outcomes in surgical patients [222–224]. Another systematic review showed that frailty is common in patients admitted to ICU and is associated with higher hospital and long-term mortality [225]. A recent scoping review focused on frailty and acute care literature that was published between 2005 and 2015 in two databases and similarly with our review recommended that future research should focus on experimental studies [226].

Strengths of this study include an in-depth search strategy and very broad scope. We included all acute care specialties and synthesized the findings based on that. Due to this, clinicians and decisions makers will be able to review specific evidence pertaining directly to their specialty. We also involved reviewers with fluency in all relevant languages which allowed us to map and synthesize all the available literature around frailty in acute care. Finally, we did a detailed search for evidence related to EMS care which usually is excluded from frailty reviews. EMS frailty screening by paramedics could facilitate referral or transport to the most appropriate service as paramedics are in a unique position to document the living conditions and function of an older person within their own home [227].

Moreover, the purpose of this review was to highlight the current gaps in frailty research. We found that non-frailty tools were commonly used to identify frailty. Established frailty tools better capture the multidimensional nature of frailty than unidimensional non-frailty tools. We also found that frailty was rarely used in experimental and qualitative articles and was rarely used as an outcome measure. It was mostly used within geriatrics, emergency department, general medicine, and cardiology; information for other medical specialties is lacking. When frailty was used to predict outcomes, rarely were patient-oriented measures, such as function and quality of life included. In addition, since almost no clinical trials have been conducted with a focus on frailty, no guidelines exist on how care planning can be modified based on frailty. These research gaps need to be filled in order to implement frailty assessment and management plans in clinical practice, and to start the discussion about changes in policy.

This review highlights seven important call to action items:

- Identify participants as frail only when it has been measured.
  - ◦ In 67% of the articles the authors identified their participants as frail but did not report on how they measured frailty.
- Report details of when and who measured frailty and other details about feasibility (e.g. time to complete assessment).
  - ◦ In 13% of the articles the authors did not report when frailty was measured and in 19% authors did not report who measured frailty.
- Use established frailty tools to measure frailty.
  - ◦ Established tools were only used in 51% of the cases.
- Conduct observational studies using patient-oriented outcomes.
  - ◦ Quality of life and functional decline only accounted for 4% of outcomes examined.
- Conduct qualitative studies about frailty.
  - ◦ Only 3% of the studies (*n* = 6) were qualitative [27, 69, 88, 124, 145, 181].
- Conduct experimental studies about modifying treatment plans based on frailty level.
  - ◦ Only one prospective study modified treatment plans based on frailty [52].
- Conduct experimental studies using frailty as an outcome.
  - ◦ Only 2% of the studies (*n* = 3) were clinical trials which used frailty as an outcome measure; two of them were published protocols [118, 119, 216].

The ability of acute care settings to cope with the influx of frail older patients may be reaching a limit, and unless changes are made in its organization, it seems inevitable that care provided to the older adult will suffer. Even though we presently lack strong evidence, identifying the frail people, those at higher risk for adverse outcomes, within EMS and in-hospital settings may lead to improvements in care. The development of a routinely collected frailty measure, such as the electronic Frailty Index of The National Health Service of England [228], can facilitate frailty to be considered in patients who come to hospital. How this will affect care has yet to be determined. Currently, comprehensive geriatric assessment (which also includes patient management) is the most effective intervention for frail older patients [229] and can also impact the patient’s frailty level. For example, a primary care model focusing on comprehensive geriatric assessment and goal-setting reversed frailty levels among older patients [230].

Recognizing the value of measuring frailty may benefit the patient and the health care system alike. By identifying frail individuals, we could increase patient-centered care and have a more efficient and effective health care system. It could lead to more targeted assessments for people who need them and end the unnecessary assessments of severely frail people. Therefore, frailty can assist clinicians in identifying patients who might benefit more from innovative processes of care than from aggressive medical treatments. Also, clinicians can use the information from the frailty assessments to discuss with patients and caregivers the risks and benefits of possible treatments, which can lead to a more informed and rational shared decision.

## Conclusions

This scoping review showed that most studies were conducted in non-geriatric disciplines and identified participants as frail without measuring frailty. There was great variability in tools used to measure frailty. The more recently published studies were more likely to use established frailty tools and the most commonly used scales were the Clinical Frailty Scale, the Frailty Index, and the Frailty Phenotype. Most studies used frailty tools to predict adverse health outcomes, especially mortality and institutionalization. Overall, frailty appears to be a good predictor of adverse health outcomes.

## Additional file

Additional file 1: **Table S1.** Medline Search Strategy. **Table S2.** The 204 articles that measured frailty. **Table S3.** Descriptive characteristics of the articles which did not include a frailty measure. **Figure S1.** Proportion of articles across disciplines. A. Articles which included a frailty measure B. Articles which did not include a frailty measure. **Figure S2.** Proportion of articles by year of publication A. Articles which included a frailty measure B. Articles which did not include a frailty measure. **Figure S3.** Proportion of articles based on who assessed frailty. **Figure S4.** Proportion of articles based on type of frailty measure used and by year of publication. **Figure S5.** Number of articles for each most commonly used frailty measure. (DOCX 123 kb)
