# Supplementary material for: What do we know about frailty in the acute care setting? A scoping review
Source: BMC Geriatr. 2018 Jun 11;18:139. doi: 10.1186/s12877-018-0823-2 (PMC6000922; doi:10.1186/s12877-018-0823-2)
Supplement: Supplementary file 1 — Table S1. Medline Search Strategy. Table S2. The 204 articles that measured frailty. Table S3. Descriptive characteristics of the articles which did not include a frailty measure. Figure S1. Proportion of articles across disciplines. A. Articles which included a frailty measure B. Articles which did not include a frailty measure. Figure S2. Proportion of articles by year of publication A. Articles which included a frailty measure B. Articles which did not include a frailty measure. Figure S3. Proportion of articles based on who assessed frailty. Figure S4. Proportion of articles based on type of frailty measure used and by year of publication. Figure S5. Number of articles for each most commonly used frailty measure. (DOCX 123 kb) [file 12877_2018_823_MOESM1_ESM.docx]

Additional file 1 **Table S1 Medline Search Strategy**

| **Database** | **Keywords** |
| --- | --- |
| Medline | “Emergency Medical Services”[Mesh] OR “Emergency Medical Technicians”[Mesh] OR paramedic*[tiab] OR emergency medical service*[tiab] OR prehospital*[tiab] OR pre-hospital*[tiab] OR emergency medical technician*[tiab] OR ambulance*[tiab] OR ((technician*[tiab] OR responder*[tiab] OR service*[tiab]) AND emergency[tiab])  OR  "Emergency Service, Hospital"[Mesh] OR "Hospitalization"[Mesh] OR "Emergency Medicine"[Mesh] OR inhospital*[tiab] OR in-hospital*[tiab] OR Emergency department*[tiab] OR Emergency Service*[tiab] OR acute care[tiab] OR admission*[tiab] OR discharge*[tiab] OR hospital*[tiab] OR inpatient*[tiab] OR in-patient*[tiab]  AND  “Aged”[Majr] OR “Frail Elderly”[Majr] OR geriatric[tiab] OR older people[tiab] OR older adults[tiab] OR elderly[tiab] OR Ageing[tiab] OR aging[tiab]  AND  frail*[tw] OR prefrail*[tw] OR pre-frail*[tw] OR non-frail*[tw] OR nonfrail* [tw] |

Additional file 1 **Table S2 The 204 articles that measured frailty**

| **References** |
| --- |
| Abbatecola AM, Spazzafumo L, Corsonello A, Sirolla C, Bustacchini S, Guffanti E. Development and validation of the HOPE prognostic index on 24-month posthospital mortality and rehospitalization: Italian National Research Center on Aging (INRCA). Rejuvenation research. 2011;14(6):605-13.  Adamis D, Treloar A, Darwiche FZ, Gregson N, Macdonald AJ, Martin FC. Associations of delirium with in-hospital and in 6-months mortality in elderly medical inpatients. Age and ageing. 2007;36(6):644-9.  Adamis D, Treloar A, Martin FC, Gregson N, Hamilton G, Macdonald AJ. APOE and cytokines as biological markers for recovery of prevalent delirium in elderly medical inpatients. International journal of geriatric psychiatry. 2007;22(7):688-94.  Aitken E, Carruthers C, Gall L, Kerr L, Geddes C, Kingsmore D. Acute kidney injury: outcomes and quality of care. QJM: An International Journal of Medicine. 2013;106(4):323-32.  Andela RM, Dijkstra A, Slaets JP, Sanderman R. Prevalence of frailty on clinical wards: description and implications. International journal of nursing practice. 2010;16(1):14-9.  Andreasen J, Sørensen EE, Gobbens RJ, Lund H, Aadahl M. Danish version of the Tilburg Frailty Indicator–translation, cross-cultural adaption and validity pretest by cognitive interviewing. Archives of Gerontology and Geriatrics. 2014;59(1):32-8.  Andrew MK, Freter SH, Rockwood K. Incomplete functional recovery after delirium in elderly people: a prospective cohort study. BMC geriatrics. 2005;5(1):5.  Ansell P, Howell D, Garry A, Kite S, Munro J, Roman E, Howard M. What determines referral of UK patients with haematological malignancies to palliative care services? An exploratory study using hospital records. Palliative Medicine. 2007;21(6):487-92.  Antunes JD, Okuno MF, Lopes MC, Campanharo CR, Batista RE. Frailty assessment of elderly hospitalized at an emergency service of a university hospital. Cogitare Enferm. 2015;20(2):264-71.  Ariza‐Solé A, Formiga F, Vidán MT, Bueno H, Curós A, Aboal J, Llibre C, Rueda F, Bernal E, Cequier A. Impact of Frailty and Functional Status on Outcomes in Elderly Patients With ST‐Segment Elevation Myocardial Infarction Undergoing Primary Angioplasty: Rationale and Design of the IFFANIAM Study. Clinical cardiology. 2013;36(10):565-9.  Aronow HU, Borenstein J, Haus F, Braunstein GD, Bolton LB. Validating SPICES as a screening tool for frailty risks among hospitalized older adults. Nursing research and practice. 2014;2014.  Arora VM, Prochaska ML, Farnan JM, D'Arcy V, Michael J, Schwanz KJ, Vinci LM, Davis AM, Meltzer DO, Johnson JK. Problems after discharge and understanding of communication with their primary care physicians among hospitalized seniors: a mixed methods study. Journal of Hospital Medicine. 2010;5(7):385-91.  Auais M, Morin S, Nadeau L, Finch L, Mayo N. Changes in frailty-related characteristics of the hip fracture population and their implications for healthcare services: evidence from Quebec, Canada. Osteoporosis International. 2013;24(10):2713-24.  Bagshaw SM, Stelfox HT, Johnson JA, McDermid RC, Rolfson DB, Tsuyuki RT, Ibrahim Q, Majumdar SR. Long-term association between frailty and health-related quality of life among survivors of critical illness: a prospective multicenter cohort study. Critical care medicine. 2015;43(5):973-82.  Baillie L, Gallini A, Corser R, Elworthy G, Scotcher A, Barrand A. Care transitions for frail, older people from acute hospital wards within an integrated healthcare system in England: a qualitative case study. International Journal of Integrated Care. 2014;14(1).  Baird S, Hill L, Rybar J, Concha‐Garcia S, Coimbra R, Patrick K. Age‐related driving disorders: Screening in hospitals and outpatients settings. Geriatrics & gerontology international. 2010;10(4):288-94.  Bakker FC, Persoon A, Bredie SJ, van Haren-Willems J, Leferink VJ, Noyez L, Schoon Y, Rikkert MG. The CareWell in Hospital program to improve the quality of care for frail elderly inpatients: results of a before–after study with focus on surgical patients. The American Journal of Surgery. 2014;208(5):735-46.  Bakker FC, Persoon A, Schoon Y, Marcel GM, Rikkert O. The CareWell in Hospital questionnaire: a measure of frail elderly inpatient experiences with individualized and integrated hospital care. Journal of hospital medicine. 2014;9(5):324-9.  Bakker FC, Persoon A, Schoon Y, Rikkert O, Marcel GM. Hospital elder life program integrated in dutch hospital care: a pilot. Journal of the American Geriatrics Society. 2013;61(4):641-2.  Baldin Storti L, Coelho Fabrício-Whebe SC, Kusumota L, Partezani Rodrigues RA, Marques S. Fragilidade de idosos internados na clínica médica da unidade de emergência de um hospital geral terciário. Texto & Contexto Enfermagem. 2013;22(2).  Baldwin MR, Reid MC, Westlake AA, Rowe JW, Granieri EC, Wunsch H, Dam TT, Rabinowitz D, Goldstein NE, Maurer MS, Lederer DJ. The feasibility of measuring frailty to predict disability and mortality in older medical intensive care unit survivors. Journal of critical care. 2014;29(3):401-8.  Basic D, Shanley C. Frailty in an older inpatient population: using the clinical frailty scale to predict patient outcomes. Journal of aging and health. 2015;27(4):670-85.  Basso U, Tonti S, Bassi C, Brunello A, Pasetto LM, Scaglione D, Falci C, Beda M, Aversa SM, Stefani M, Castegnaro E. Management of Frail and Not-Frail elderly cancer patients in a hospital-based geriatric oncology program. Critical reviews in oncology/hematology. 2008;66(2):163-70.  Bennett A, Gnjidic D, Gillett M, Carroll P, Matthews S, Johnell K, Fastbom J, Hilmer S. Prevalence and impact of fall-risk-increasing drugs, polypharmacy, and drug–drug interactions in robust versus frail hospitalised falls patients: a prospective cohort study. Drugs & aging. 2014;31(3):225-32.  Bo M, Fonte G, Pivaro F, Bonetto M, Comi C, Giorgis V, Marchese L, Isaia G, Maggiani G, Furno E, Falcone Y. Prevalence of and factors associated with prolonged length of stay in older hospitalized medical patients. Geriatrics & gerontology international. 2016 ;16(3):314-21.  Bouzereau V, Le Caer F, Guardiola E, Scavennec C, Barriere JR, Chaix L, Le Caer H. Experience of multidisciplinary assessment of elderly patients with cancer in a French general hospital during 1year: A new model care study. Journal of geriatric oncology. 2013;4(4):394-401.  Brenner SS, Klotz U. P-glycoprotein function in the elderly. European journal of clinical pharmacology. 2004;60(2):97-102.  Brunet NM, Panicot JE, Sevilla-Sánchez D, Novellas JA, Jané CC, Roset JA, Gómez-Batiste X. A patient-centered prescription model assessing the appropriateness of chronic drug therapy in older patients at the end of life. European Geriatric Medicine. 2015;6(6):565-9.  Buck HG, Riegel B. The impact of frailty on health related quality of life in heart failure. European Journal of Cardiovascular Nursing. 2011;10(3):159-66.  Bugat ME, Gerard S, Balardy L, Beyne-Rauzy O, Boussier N, Perrin A, Oustric S, Vellas B, Nourhashemi F. Impact of an oncogeriatric consulting team on therapeutic decision-making. The journal of nutrition, health & aging. 2013;17(5):473.  Bustacchini S, Abbatecola AM, Bonfigli AR, Chiatti C, Corsonello A, Di Stefano G, Galeazzi R, Fabbietti P, Lisa R, Guffanti EE, Provinciali M. The Report-AGE project: a permanent epidemiological observatory to identify clinical and biological markers of health outcomes in elderly hospitalized patients in Italy. Aging clinical and experimental research. 2015;27(6):893-901.  Buttery AK, Martin FC. Knowledge, attitudes and intentions about participation in physical activity of older post-acute hospital inpatients. Physiotherapy. 2009;95(3):192-8.  Capan M, Ivy JS, Rohleder T, Hickman J, Huddleston JM. Individualizing and optimizing the use of early warning scores in acute medical care for deteriorating hospitalized patients. Resuscitation. 2015;93:107-12.  Chouliara Z, Kearney N, Worth A, Stott D. Challenges in conducting research with hospitalized older people with cancer: drawing from the experience of an ongoing interview‐based project. European journal of cancer care. 2004;13(5):409-15.  Christodoulidis G, Yu J, Kini A, Dangas GD, Baber U, Melissa A, Sartori S, Theodoropoulos K, Bhat A, Kovacic J, Moreno P. Gender-specific outcomes after balloon aortic valvuloplasty: Inhospital and long-term outcomes. American heart journal. 2015;170(1):180-6.  Cohen HJ, Feussner JR, Weinberger M, Carnes M, Hamdy RC, Hsieh F, Phibbs C, Courtney D, Lyles KW, May C, McMurtry C. A controlled trial of inpatient and outpatient geriatric evaluation and management. New England Journal of Medicine. 2002;346(12):905-12.  Cohen M. Research assessment of elder neglect and its risk factors in a hospital setting. Internal medicine journal. 2008;38(9):704-7.  Compté N, Bailly B, De Breucker S, Goriely S, Pepersack T. Study of the association of total and differential white blood cell counts with geriatric conditions, cardio-vascular diseases, seric IL-6 levels and telomere length. Experimental gerontology. 2015;61:105-12.  Compte N, Boudjeltia KZ, Vanhaeverbeek M, De Breucker S, Tassignon J, Trelcat A, Pepersack T, Goriely S. Frailty in old age is associated with decreased interleukin-12/23 production in response to toll-like receptor ligation. PloS one. 2013;8(6):e65325.  Conroy S, Dowsing T. The ability of frailty to predict outcomes in older people attending an acute medical unit. Acute medicine. 2013;12(2):74-6.  Conroy SP, Dowsing T, Reid J, Hsu R. Understanding readmissions: An in-depth review of 50 patients readmitted back to an acute hospital within 30days. European Geriatric Medicine. 2013;4(1):25-7.  Copeland LA, Zeber JE, Rosenheck RA, Miller AL. Unforeseen inpatient mortality among veterans with schizophrenia. Medical care. 2006;44(2):110-6.  Corona G, Polesel J, Fratino L, Miolo G, Rizzolio F, Crivellari D, Addobbati R, Cervo S, Toffoli G. Metabolomics biomarkers of frailty in elderly breast cancer patients. Journal of cellular physiology. 2014;229(7):898-902.  Crehan F, O'Shea D, Ryan JM, Horgan F. A profile of elderly fallers referred for physiotherapy in the emergency department of a Dublin teaching hospital. Irish Medical Journal. 2013; 106(6):173-6.  Dai YT, Wu SC, Weng R. Unplanned hospital readmission and its predictors in patients with chronic conditions. Journal of the Formosan Medical Association. 2002;101(11):779-85.  Dalleur O, Boland B, Losseau C, Henrard S, Wouters D, Speybroeck N, Degryse JM, Spinewine A. Reduction of potentially inappropriate medications using the STOPP criteria in frail older inpatients: a randomised controlled study. Drugs & aging. 2014;31(4):291-8.  Dalleur O, Spinewine A, Henrard S, Losseau C, Speybroeck N, Boland B. Inappropriate prescribing and related hospital admissions in frail older persons according to the STOPP and START criteria. Drugs & aging. 2012;29(10):829-37.  De Brauwer I, Lepage S, Yombi JC, Cornette P, Boland B. Prediction of risk of in-hospital geriatric complications in older patients with hip fracture. Aging Clin Exp Res. 2012;24(1):62-7.  De Breucker S, Herzog G, Pepersack T. Could Geriatric Characteristics Explain the Under-Prescription of Anticoagulation Therapy for Older Patients Admitted with Atrial Fibrillation? Drugs & aging. 2010;27(10):807-13.  Dellasega CA, Zerbe TM. A multimethod study of advanced practice nurse postdischarge care. Clinical excellence for nurse practitioners: the international journal of NPACE. 2000;4(5):286-93.  Denoël P, Vanderstraeten J, Mols P, Pepersack T. Could some geriatric characteristics hinder the prescription of anticoagulants in atrial fibrillation in the elderly?. Journal of aging research. 2014;2014.  Dent E, Perez-Zepeda M. Comparison of five indices for prediction of adverse outcomes in hospitalised Mexican older adults: a cohort study. Archives of gerontology and geriatrics. 2015;60(1):89-95.  Dorner TE, Lackinger C, Haider S, Luger E, Kapan A, Luger M, Schindler KE. Nutritional intervention and physical training in malnourished frail community-dwelling elderly persons carried out by trained lay “buddies”: study protocol of a randomized controlled trial. BMC Public Health. 2013;13(1):1232.  Dorner TE, Luger E, Tschinderle J, Stein KV, Haider S, Kapan A, Lackinger C, Schindler KE. Association between nutritional status (MNA®-SF) and frailty (SHARE-FI) in acute hospitalised elderly patients. The journal of nutrition, health & aging. 2014;18(3):264-9.  Dramé M, Novella JL, Jolly D, Lanièce I, Somme D, Heitz D, Gauvain JB, Voisin T, De Wazières B, Gonthier R, Jeandel C. Rapid cognitive decline, one-year institutional admission and one-year mortality: analysis of the ability to predict and inter-tool agreement of four validated clinical frailty indexes in the SAFEs cohort. The journal of nutrition, health & aging. 2011;15(8):699-705.  Duffy SA, Copeland LA, Hopp FP, Zalenski RJ. Diagnostic classifications and resource utilization of decedents served by the Department of Veterans Affairs. Journal of palliative medicine. 2007;10(5):1137-45.  Dwyer JG, Reynoso JF, Seevers GA, Schmid KK, Muralidhar P, Konigsberg B, Lynch TG, Johanning JM. Assessing preoperative frailty utilizing validated geriatric mortality calculators and their association with postoperative hip fracture mortality risk. Geriatric orthopaedic surgery & rehabilitation. 2014;5(3):109-15.  Eeles EM, White SV, O'mahony SM, Bayer AJ, Hubbard RE. The impact of frailty and delirium on mortality in older inpatients. Age and ageing. 2012 Mar 4;41(3):412-6.  Ekdahl AW, Andersson L, Friedrichsen M. “They do what they think is the best for me.” Frail elderly patients’ preferences for participation in their care during hospitalization. Patient education and counseling. 2010;80(2):233-40.  Ekerstad N, Swahn E, Janzon M, Alfredsson J, Löfmark R, Lindenberger M, Carlsson P. Frailty is independently associated with short-term outcomes for elderly patients with non–ST-segment elevation myocardial infarction. Circulation. 2011; 124(22):2397-404.  Eklund K, Wilhelmson K, Gustafsson H, Landahl S, Dahlin-Ivanoff S. One-year outcome of frailty indicators and activities of daily living following the randomised controlled trial;“Continuum of care for frail older people”. BMC geriatrics. 2013;13(1):76.  El-Sharkawy AM, Watson P, Neal KR, Ljungqvist O, Maughan RJ, Sahota O, Lobo DN. Hydration and outcome in older patients admitted to hospital (The HOOP prospective cohort study). Age and ageing. 2015;44(6):943-7.  Ertel KA, Glymour MM, Glass TA, Berkman LF. Frailty modifies effectiveness of psychosocial intervention in recovery from stroke. Clinical rehabilitation. 2007;21(6):511-22.  Evans SJ, Sayers M, Mitnitski A, Rockwood K. The risk of adverse outcomes in hospitalized older patients in relation to a frailty index based on a comprehensive geriatric assessment. Age and ageing. 2014;43(1):127-32.  Fairchild B, Webb TP, Xiang Q, Tarima S, Brasel KJ. Sarcopenia and frailty in elderly trauma patients. World journal of surgery. 2015;39(2):373-9.  Farhat JS, Velanovich V, Falvo AJ, Horst HM, Swartz A, Patton Jr JH, Rubinfeld IS. Are the frail destined to fail? Frailty index as predictor of surgical morbidity and mortality in the elderly. Journal of Trauma and Acute Care Surgery. 2012;72(6):1526-31.  Ferguson MK, Thompson K, Huisingh-Scheetz M, Farnan J, Hemmerich JA, Slawinski K, Acevedo J, Lee SM, Rojnica M, Small S. Thoracic surgeons' perception of frail behavior in videos of standardized patients. PloS one. 2014;9(6):e98654.  Ferguson MK, Thompson K, Huisingh-Scheetz M, Farnan J, Hemmerich J, Acevedo J, Small S. The Impact of a Frailty Education Module on Surgical Resident Estimates of Lobectomy Risk. The Annals of thoracic surgery. 2015;100(1):235-41.  Fisher C, Karalapillai DK, Bailey M, Glassford NG, Bellomo R, Jones D. Predicting intensive care and hospital outcome with the Dalhousie Clinical Frailty Scale: a pilot assessment. Anaesthesia & Intensive Care. 2015;43(3).  Fontana L, Addante F, Copetti M, Paroni G, Fontana A, Sancarlo D, Pellegrini F, Ferrucci L, Pilotto A. Identification of a metabolic signature for multidimensional impairment and mortality risk in hospitalized older patients. Aging cell. 2013;12(3):459-66.  Forti P, Maioli F, Zagni E, Lucassenn T, Montanari L, Maltoni B, Pirazzoli GL, Bianchi G, Zoli M. The physical phenotype of frailty for risk stratification of older medical inpatients. The journal of nutrition, health & aging. 2014;18(10):912-8.  Fracchia S, Grasso A, Pagani M, Corsini C, Cerina G, Ghirmai S, Bernardini B, Berra C, Badalamenti S, Campanati P, Boncinelli S. Il paziente anziano nel setting di cura per acuti: ruolo di uno strumento di assessment rapido multidimensionale nell’identificazione dei pazienti a rischio. G Gerontol. 2011;59:130-9.  Frank C, Touw M, Suurdt J, Jiang X, Wattam P, Heyland DK. Optimizing end-of-life care on medical clinical teaching units using the CANHELP questionnaire and a nurse facilitator: a feasibility study. CJNR (Canadian Journal of Nursing Research). 2012;44(1):40-58.  Frew E, Sequeira J, Cant R. Nutrition screening process for patients in an acute public hospital servicing an elderly, culturally diverse population. Nutrition & Dietetics. 2010;67(2):71-6.  Gharacholou SM, Sloane R, Cohen HJ, Schmader KE. Geriatric inpatient units in the care of hospitalized frail adults with a history of heart failure. International Journal of Gerontology. 2012;6(2):112-6.  Goldstein J, Hubbard RE, Moorhouse P, Andrew MK, Mitnitski A, Rockwood K. The validation of a care partner-derived frailty index based upon comprehensive geriatric assessment (CP-FI-CGA) in emergency medical services and geriatric ambulatory care. Age and ageing. 2014;44(2):327-30.  Graham MM, Galbraith PD, O'Neill D, Rolfson DB, Dando C, Norris CM. Frailty and outcome in elderly patients with acute coronary syndrome. Canadian Journal of Cardiology. 2013;29(12):1610-5.  Gregersen M, Borris LC, Damsgaard EM. Blood transfusion and overall quality of life after hip fracture in frail elderly patients—the transfusion requirements in frail elderly randomized controlled trial. Journal of the American Medical Directors Association. 2015;16(9):762-6.  Gregersen M, Borris LC, Damsgaard EM. Postoperative blood transfusion strategy in frail, anemic elderly patients with hip fracture: the TRIFE randomized controlled trial. Acta orthopaedica. 2015;86(3):363-72.  Gregersen M, Damsgaard EM, Borris LC. Blood transfusion and risk of infection in frail elderly after hip fracture surgery: the TRIFE randomized controlled trial. European Journal of Orthopaedic Surgery & Traumatology. 2015;25(6):1031-8.  Grossman D, Rootenberg M, Perri GA, Yogaparan T, DeLeon M, Calabrese S, Grief CJ, Moore J, Gill A, Stilos K, Daines P. Enhancing communication in end‐of‐life care: a clinical tool translating between the Clinical Frailty Scale and the Palliative Performance Scale. Journal of the American Geriatrics Society. 2014;62(8):1562-7.  Guerra IC, Ramos-Cerqueira AT. Risco de hospitalizações repetidas em idosos usuários de um centro de saúde escola. Cadernos de Saúde Pública. 2007:585-92.  Hajjar ER, Hanlon JT, Sloane RJ, Lindblad CI, Pieper CF, Ruby CM, Branch LC, Schmader KE. Unnecessary drug use in frail older people at hospital discharge. Journal of the American Geriatrics Society. 2005;53(9):1518-23.  Hanlon JT, Artz MB, Pieper CF, Lindblad CI, Sloane RJ, Ruby CM, Schmader KE. Inappropriate medication use among frail elderly inpatients. Annals of Pharmacotherapy. 2004;38(1):9-14.  Hanlon JT, Maher RL, Lindblad CI, Ruby CM, Twersky J, Cohen HJ, Schmader KE. Comparison of methods for detecting potential adverse drug events in frail elderly inpatients and outpatients. American journal of health-system pharmacy. 2001;58(17):1622-6.  Hanlon JT, Pieper CF, Hajjar ER, Sloane RJ, Lindblad CI, Ruby CM, Schmader KE. Incidence and predictors of all and preventable adverse drug reactions in frail elderly persons after hospital stay. The Journals of Gerontology Series A: Biological Sciences and Medical Sciences. 2006;61(5):511-5.  Hansen T, Lambert HC, Faber J. Ingestive Skill Difficulties are Frequent Among Acutely-Hospitalized Frail Elderly Patients, and Predict Hospital Outcomes. Physical & Occupational Therapy In Geriatrics. 2012;30(4):271-87.  Harvey P, Storer M, Berlowitz DJ, Jackson B, Hutchinson A, Lim WK. Feasibility and impact of a post–discharge geriatric evaluation and management service for patients from residential care: the Residential Care Intervention Program in the Elderly (RECIPE). BMC geriatrics. 2014;14(1):48.  Heim N, van Fenema EM, Weverling-Rijnsburger AW, Tuijl JP, Jue P, Oleksik AM, Verschuur MJ, Haverkamp JS, Blauw GJ, van der Mast RC, Westendorp RG. Optimal screening for increased risk for adverse outcomes in hospitalised older adults. Age and ageing. 2014;44(2):239-44.  Hendrix CC, Hastings SN, Van Houtven C, Steinhauser K, Chapman J, Ervin T, Sanders L, Weinberger M. Pilot study: Individualized training for caregivers of hospitalized older veterans. Nursing research. 2011;60(6):436-41.  Herrmann FR, Osiek A, Cos M, Michel JP, Robine JM. Frailty judgment by hospital team members: degree of agreement and survival prediction. Journal of the American Geriatrics Society. 2005;53(5):916-7.  Heyland D, Cook D, Bagshaw SM, Garland A, Stelfox HT, Mehta S, Dodek P, Kutsogiannis J, Burns K, Muscedere J, Turgeon AF. The very elderly admitted to ICU: a quality finish? Critical care medicine. 2015;43(7):1352-60.  Heyland DK, Dodek P, Mehta S, Cook D, Garland A, Stelfox HT, Bagshaw SM, Kutsogiannis DJ, Burns K, Muscedere J, Turgeon AF. Admission of the very elderly to the intensive care unit: Family members’ perspectives on clinical decision-making from a multicenter cohort study. Palliative medicine. 2015;29(4):324-35.  Heyland DK, Garland A, Bagshaw SM, Cook D, Rockwood K, Stelfox HT, Dodek P, Fowler RA, Turgeon AF, Burns K, Muscedere J. Recovery after critical illness in patients aged 80 years or older: a multi-center prospective observational cohort study. Intensive care medicine. 2015;41(11):1911-20.  Hignett S, Sands G, Griffiths P. In-patient falls: what can we learn from incident reports?. Age and ageing. 2013 Jul 1;42(4):527-31.  Hii TB, Lainchbury JG, Bridgman PG. Frailty in acute cardiology: comparison of a quick clinical assessment against a validated frailty assessment tool. Heart, Lung and Circulation. 2015;24(6):551-6.  Hill L, Rybar J, Baird S, Concha-Garcia S, Coimbra R, Patrick K. Road safe seniors: Screening for age-related driving disorders in inpatient and outpatient settings. Journal of safety research. 2011;42(3):165-9.  Hilmer SN, Perera V, Mitchell S, Murnion BP, Dent J, Bajorek B, Matthews S, Rolfson DB. The assessment of frailty in older people in acute care. Australasian journal on ageing. 2009;28(4):182-8.  Hope AA, Gong MN, Guerra C, Wunsch H. Frailty before critical illness and mortality for elderly Medicare beneficiaries. Journal of the American Geriatrics Society. 2015;63(6):1121-8.  Housley BC, Stawicki SP, Evans DC, Jones C. Comorbidity-polypharmacy score predicts readmission in older trauma patients. Journal of surgical research. 2015;199(1):237-43.  Hubbard RE, Eeles EM, Rockwood MR, Fallah N, Ross E, Mitnitski A, Rockwood K. Assessing balance and mobility to track illness and recovery in older inpatients. Journal of general internal medicine. 2011;26(12):1471-8.  Huijberts S, Buurman BM, de Rooij SE. End-of-life care during and after an acute hospitalization in older patients with cancer, end-stage organ failure, or frailty: A sub-analysis of a prospective cohort study. Palliative medicine. 2016;30(1):75-82.  Hunt K, Walsh B, Voegeli D, Roberts H. Reducing avoidable hospital admission in older people: Health status, frailty and predicting risk of ill-defined conditions diagnoses in older people admitted with collapse. Archives of gerontology and geriatrics. 2013;57(2):172-6.  Ijkema R, Langelaan M, Van de Steeg L, Wagner C. What impedes and what facilitates a quality improvement project for older hospitalized patients?. International journal for quality in health care. 2013;26(1):41-8.  Joosten E, Demuynck M, Detroyer E, Milisen K. Prevalence of frailty and its ability to predict in hospital delirium, falls, and 6-month mortality in hospitalized older patients. BMC geriatrics. 2014;14(1):1.  Joseph B, Pandit V, Khalil M, Kulvatunyou N, Zangbar B, Friese RS, Mohler MJ, Fain MJ, Rhee P. Managing Older Adults with Ground‐Level Falls Admitted to a Trauma Service: The Effect of Frailty. Journal of the American Geriatrics Society. 2015;63(4):745-9.  Joseph B, Pandit V, Rhee P, Aziz H, Sadoun M, Wynne J, Tang A, Kulvatunyou N, O’Keeffe T, Fain MJ, Friese RS. Predicting hospital discharge disposition in geriatric trauma patients: is frailty the answer? Journal of Trauma and Acute Care Surgery. 2014;76(1):196-200.  Joseph B, Pandit V, Zangbar B, Kulvatunyou N, Hashmi A, Green DJ, O’Keeffe T, Tang A, Vercruysse G, Fain MJ, Friese RS. Superiority of frailty over age in predicting outcomes among geriatric trauma patients: a prospective analysis. JAMA surgery. 2014;149(8):766-72.  Joseph B, Pandit V, Zangbar B, Kulvatunyou N, Tang A, O'Keeffe T, Green DJ, Vercruysse G, Fain MJ, Friese RS, Rhee P. Validating trauma-specific frailty index for geriatric trauma patients: a prospective analysis. Journal of the American College of Surgeons. 2014;219(1):10-7.  Kaiser RM, Schmader KE, Pieper CF, Lindblad CI, Ruby CM, Hanlon JT. Therapeutic failure-related hospitalisations in the frail elderly. Drugs & aging. 2006;23(7):579-86.  Kenig J, Zychiewicz B, Olszewska U, Barczynski M, Nowak W. Six screening instruments for frailty in older patients qualified for emergency abdominal surgery. Archives of gerontology and geriatrics. 2015;61(3):437-42.  Khandelwal D, Goel A, Kumar U, Gulati V, Narang R, Dey AB. Frailty is associated with longer hospital stay and increased mortality in hospitalized older patients. The journal of nutrition, health & aging. 2012;1:1-4.  Kimmel LA, Holland AE, Simpson PM, Edwards ER, Gabbe BJ. Validating a simple discharge planning tool following hospital admission for an isolated lower limb fracture. Physical therapy. 2014;94(7):1005-13.  Kistler EA, Nicholas JA, Kates SL, Friedman SM. Frailty and short-term outcomes in patients with hip fracture. Geriatric orthopaedic surgery & rehabilitation. 2015;6(3):209-14.  Klepin HD, Geiger AM, Tooze JA, Kritchevsky SB, Williamson JD, Ellis LR, Levitan D, Pardee TS, Isom S, Powell BL. The feasibility of inpatient geriatric assessment for older adults receiving induction chemotherapy for acute myelogenous leukemia. Journal of the American Geriatrics Society. 2011;59(10):1837-46.  Krishnan M, Beck S, Havelock W, Eeles E, Hubbard RE, Johansen A. Predicting outcome after hip fracture: using a frailty index to integrate comprehensive geriatric assessment results. Age and ageing. 2013;43(1):122-6.  Le Maguet P, Roquilly A, Lasocki S, Asehnoune K, Carise E, Saint Martin M, Mimoz O, Le Gac G, Somme D, Cattenoz C, Feuillet F. Prevalence and impact of frailty on mortality in elderly ICU patients: a prospective, multicenter, observational study. Intensive care medicine. 2014;40(5):674-82.  Lee J, Sirois MJ, Moore L, Perry J, Daoust R, Griffith L, Worster A, Lang E, Emond M. Return to the ED and hospitalisation following minor injuries among older persons treated in the emergency department: predictors among independent seniors within 6 months. Age and ageing. 2015;44(4):624-9.  Lefebvre MC, St-Onge M, Glazer-Cavanagh M, Bell L, Nguyen JN, Nguyen PV, Tannenbaum C. The effect of bleeding risk and frailty status on anticoagulation patterns in octogenarians with atrial fibrillation: the FRAIL-AF study. Canadian Journal of Cardiology. 2016;32(2):169-76.  Leung DY, Lee DT, Lee IF, Lam LW, Lee SW, Chan MW, Lam YM, Leung SH, Chiu PC, Ho NK, Ip MF. The effect of a virtual ward program on emergency services utilization and quality of life in frail elderly patients after discharge: a pilot study. Clinical interventions in aging. 2015;10:413.  Li Y, Zou Y, Wang S, Li J, Jing X, Yang M, Wang L, Cao L, Yang X, Xu L, Dong B. A pilot study of the FRAIL scale on predicting outcomes in Chinese elderly people with type 2 diabetes. Journal of the American Medical Directors Association. 2015;16(8):714-e7.  Lichtman JH, Krumholz HM, Wang Y, Radford MJ, Brass LM. Risk and predictors of stroke after myocardial infarction among the elderly. Circulation. 2002;105(9):1082-7.  Lindberg M, Saltvedt I, Sletvold O, Bjerve KS. Long-chain n− 3 fatty acids and mortality in elderly patients. The American journal of clinical nutrition. 2008;88(3):722-9.  Lindblad CI, Artz MB, Pieper CF, Sloane RJ, Hajjar ER, Ruby CM, Schmader KE, Hanlon JT. Potential Drug—Disease Interactions in Frail, Hospitalized Elderly Veterans. Annals of Pharmacotherapy. 2005;39(3):412-7.  Lindblad CI, Hanlon JT, Gross CR, Sloane RJ, Pieper CF, Hajjar ER, Ruby CM, Schmader KE, Panel MC. Clinically important drug-disease interactions and their prevalence in older adults. Clinical therapeutics. 2006;28(8):1133-43.  Lindhardt T, Nyberg P, Hallberg IR. Collaboration between relatives of elderly patients and nurses and its relation to satisfaction with the hospital care trajectory. Scandinavian journal of caring sciences. 2008;22(4):507-19.  Lisiecki J, Zhang P, Wang L, Rinkinen J, De La Rosa S, Enchakalody B, Brownley RC, Wang SC, Buchman SR, Levi B. Morphomic Measurement of the Temporalis Muscle and Zygomatic Bone as Novel Predictors of Hospital-Based Clinical Outcomes in Patients with Mandible Fracture. Journal of Craniofacial Surgery. 2013;24(5):1577-81.  Lococo F, Cesario A, Margaritora S, Nachira D, Leuzzi G, Porziella V, Meacci E, Vita ML, Congedo MT, Granone P. Clinical effect of bovine pericardial strips on air leak after stapled pulmonary resection in" frail" patients: early results. Minerva chirurgica. 2012;67(1):87-94.  Ma HM, Yu RH, Woo J. Recurrent hospitalisation with pneumonia is associated with higher 1‐year mortality in frail older people. Internal medicine journal. 2013;43(11):1210-5.  MacIntyre CR, Ridda I, Gao Z, Moa AM, McIntyre PB, Sullivan JS, Jones TR, Hayen A, Lindley RI. A randomized clinical trial of the immunogenicity of 7-valent pneumococcal conjugate vaccine compared to 23-valent polysaccharide vaccine in frail, hospitalized elderly. PLoS One. 2014;9(4):e94578.  Maes F, Dalleur O, Henrard S, Wouters D, Scavée C, Spinewine A, Boland B. Risk scores and geriatric profile: can they really help us in anticoagulation decision making among older patients suffering from atrial fibrillation?. Clinical interventions in aging. 2014;9:1091.  Martínez-Velilla N, Casas-Herrero A, Zambom-Ferraresi F, Suárez N, Alonso-Renedo J, Contín KC, de Asteasu ML, Echeverria NF, Lázaro MG, Izquierdo M. Functional and cognitive impairment prevention through early physical activity for geriatric hospitalized patients: study protocol for a randomized controlled trial. BMC geriatrics. 2015;15(1):112.  Massa E, Madeddu C, Astara G, Pisano M, Spiga C, Tanca FM, Sanna E, Puddu I, Patteri E, Lamonica G, Deiana L. An attempt to correlate a “Multidimensional Geriatric Assessment”(MGA), treatment assignment and clinical outcome in elderly cancer patients: results of a phase II open study. Critical reviews in oncology/hematology. 2008;66(1):75-83.  Masud D, Norton S, Smailes S, Shelley O, Philp B, Dziewulski P. The use of a frailty scoring system for burns in the elderly. Burns. 2013;39(1):30-6.  Matsuzawa T, Sakurai T, Kuranaga M, Endo H, Yokono K. Predictive factors for hospitalized and institutionalized care-giving of the aged patients with diabetes mellitus in Japan. Kobe J. Med. Sci. 2010;56(4):E173-83.  Matsuzawa Y, Konishi M, Akiyama E, Suzuki H, Nakayama N, Kiyokuni M, Sumita S, Ebina T, Kosuge M, Hibi K, Tsukahara K. Association between gait speed as a measure of frailty and risk of cardiovascular events after myocardial infarction. Journal of the American College of Cardiology. 2013;61(19):1964-72.  Maxwell CA, Dietrich MS, Minnick AF, Mion LC. Preinjury Physical Function and Frailty in Injured Older Adults: Self‐Versus Proxy Responses. Journal of the American Geriatrics Society. 2015;63(7):1443-7.  Maxwell CA, Mion LC, Mukherjee K, Dietrich MS, Minnick A, May A, Miller RS. Feasibility of screening for preinjury frailty in hospitalized injured older adults. Journal of trauma and acute care surgery. 2015;78(4):844-51.  Maxwell CA. Screening hospitalized injured older adults for cognitive impairment and pre-injury functional impairment. Applied nursing research. 2013;26(3):146-50.  Mitchell SJ, Hilmer SN, Murnion BP, Matthews S. Hepatotoxicity of therapeutic short‐course paracetamol in hospital inpatients: impact of ageing and frailty. Journal of clinical pharmacy and therapeutics. 2011;36(3):327-35.  Moorhouse P, Mallery LH. Palliative and therapeutic harmonization: A model for appropriate decision‐making in frail older adults. Journal of the American Geriatrics Society. 2012;60(12):2326-32.  Myers V, Broday DM, Steinberg DM, Drory Y, Gerber Y. Exposure to particulate air pollution and long-term incidence of frailty after myocardial infarction. Annals of epidemiology. 2013;23(7):395-400.  Njemini R, Bautmans I, Onyema OO, Van Puyvelde K, Demanet C, Mets T. Circulating heat shock protein 70 in health, aging and disease. BMC immunology. 2011;12(1):24.  Oliveira DR, Bettinelli LA, Pasqualotti A, Corso D, Brock F, Erdmann AL. Prevalence of frailty syndrome in old people in a hospital institution. Revista latino-americana de enfermagem. 2013;21(4):891-8.  Oo MT, Tencheva A, Khalid N, Chan YP, Ho SF. Assessing frailty in the acute medical admission of elderly patients. The journal of the Royal College of Physicians of Edinburgh. 2012;43(4):301-8.  Ormerod JO, Ramcharitar S. Does specific interventional risk scoring better predict mortality than comorbidity in nonagenerians undergoing coronary angioplasty?. Cardiovascular Revascularization Medicine. 2014;15(4):258-60.  Parmar KR, Xiu PY, Chowdhury MR, Patel E, Cohen M. In-hospital treatment and outcomes of heart failure in specialist and non-specialist services: a retrospective cohort study in the elderly. Open heart. 2015;2(1):e000095.  Patel KV, Brennan KL, Brennan ML, Jupiter DC, Shar A, Davis ML. Association of a modified frailty index with mortality after femoral neck fracture in patients aged 60 years and older. Clinical Orthopaedics and Related Research®. 2014;472(3):1010-7.  Pepersack T. Minimum geriatric screening tools to detect common geriatric problems. The journal of nutrition, health & aging. 2008;12(5):348-52.  Perera V, Bajorek BV, Matthews S, Hilmer SN. The impact of frailty on the utilisation of antithrombotic therapy in older patients with atrial fibrillation. Age and ageing. 2009;38(2):156-62.  Phillips AC, Upton J, Duggal NA, Carroll D, Lord JM. Depression following hip fracture is associated with increased physical frailty in older adults: the role of the cortisol: dehydroepiandrosterone sulphate ratio. BMC geriatrics. 2013;13(1):60.  Pilotto A, Rengo F, Marchionni N, Sancarlo D, Fontana A, Panza F, Ferrucci L, FIRI-SIGG study group. Comparing the prognostic accuracy for all-cause mortality of frailty instruments: a multicentre 1-year follow-up in hospitalized older patients. PloS one. 2012;7(1):e29090.  Pitkala KH, Laurila JV, Strandberg TE, Tilvis RS. Prognostic significance of delirium in frail older people. Dementia and geriatric cognitive disorders. 2005;19(2-3):158-63.  Polidoro A, Stefanelli F, Ciacciarelli M, Pacelli A, Di Sanzo D, Alessandri C. Frailty in patients affected by atrial fibrillation. Archives of gerontology and geriatrics. 2013;57(3):325-7.  Ponzetti A, Lista P, Pagano E, Demichelis MM, Ciuffreda L, Ciccone G. Role of multidimensional assessment of frailty in predicting short-term outcomes in hospitalized cancer patients: results of a prospective cohort study. Tumori. 2014;100(1):91-6.  Popejoy L. Participation of elder persons, families, and health care teams in hospital discharge destination decisions. Applied Nursing Research. 2011;24(4):256-62.  Popejoy LL. Complexity of family caregiving and discharge planning. Journal of family nursing. 2011;17(1):61-81.  Poudel A, Peel NM, Nissen L, Mitchell C, Gray LC, Hubbard RE. Potentially inappropriate prescribing in older patients discharged from acute care hospitals to residential aged care facilities. Annals of Pharmacotherapy. 2014;48(11):1425-33.  Provencher V, Sirois MJ, Ouellet MC, Camden S, Neveu X, Allain‐Boulé N, Emond M. Decline in activities of daily living after a visit to a Canadian emergency department for minor injuries in independent older adults: are frail older adults with cognitive impairment at greater risk?. Journal of the American Geriatrics Society. 2015;63(5):860-8.  Pugh JA, Wang CP, Espinoza SE, Noël PH, Bollinger M, Amuan M, Finley E, Pugh MJ. Influence of Frailty‐Related Diagnoses, High‐Risk Prescribing in Elderly Adults, and Primary Care Use on Readmissions in Fewer than 30 Days for Veterans Aged 65 and Older. Journal of the American Geriatrics Society. 2014;62(2):291-8.  Purser JL, Kuchibhatla MN, Fillenbaum GG, Harding T, Peterson ED, Alexander KP. Identifying frailty in hospitalized older adults with significant coronary artery disease. Journal of the American Geriatrics Society. 2006;54(11):1674-81.  Rao AV, Hsieh F, Feussner JR, Cohen HJ. Geriatric evaluation and management units in the care of the frail elderly cancer patient. The Journals of Gerontology Series A: Biological Sciences and Medical Sciences. 2005;60(6):798-803.  Rao MY, Rao TS, Narayanaswamy RK. Study of hypothalamo pituitary adrenal axis in frail elderly subjects. J Assoc Physicians India. 2012;60:31-4.  Ridda I, Lindley R, MacIntyre RC. The challenges of clinical trials in the exclusion zone: the case of the frail elderly. Australasian Journal on Ageing. 2008;27(2):61-6.  Ritt M, Schwarz C, Kronawitter V, Delinic A, Bollheimer LC, Gassmann KG, Sieber CC. Analysis of Rockwood et al’s Clinical Frailty Scale and Fried et al’s frailty phenotype as predictors of mortality and other clinical outcomes in older patients who were admitted to a geriatric ward. The journal of nutrition, health & aging. 2015;19(10):1043-8.  Rockwood K, Rockwood MR, Andrew MK, Mitnitski A. Reliability of the hierarchical assessment of balance and mobility in frail older adults. Journal of the American Geriatrics Society. 2008;56(7):1213-7.  Rodríguez-Molinero A, López-Diéguez M, Tabuenca AI, de la Cruz JJ, Banegas JR. Functional assessment of older patients in the emergency department: comparison between standard instruments, medical records and physicians' perceptions. BMC geriatrics. 2006;6(1):13.  Rose M, Pan H, Levinson MR, Staples M. Can frailty predict complicated care needs and length of stay?. Internal medicine journal. 2014;44(8):800-5.  Rosted E, Schultz M, Dynesen H, Dahl M, Sørensen M, Sanders S. The Identification of Seniors at Risk screening tool is useful for predicting acute readmissions. Dan Med J. 2014;61(5):A4828.  Ryan T, Ingleton C, Gardiner C, Parker C, Gott M, Noble B. Symptom burden, palliative care need and predictors of physical and psychological discomfort in two UK hospitals. BMC palliative care. 2013;12(1):11.  Saltvedt I, Jordhøy M, Mo ES, Fayers P, Kaasa S, Sletvold O. Randomised trial of in-hospital geriatric intervention: impact on function and morale. Gerontology. 2006;52(4):223-30.  Saltvedt I, Mo ES, Fayers P, Kaasa S, Sletvold O. Reduced mortality in treating acutely sick, frail older patients in a geriatric evaluation and management unit. A prospective randomized trial. Journal of the American Geriatrics Society. 2002;50(5):792-8.  Saltvedt I, Saltnes T, Mo ES, Fayers P, Kaasa S, Sletvold O. Acute geriatric intervention increases the number of patients able to live at home. A prospective randomized study. Aging clinical and experimental research. 2004;16(4):300-6.  Saltvedt I, Spigset O, Ruths S, Fayers P, Kaasa S, Sletvold O. Patterns of drug prescription in a geriatric evaluation and management unit as compared with the general medical wards: a randomised study. European journal of clinical pharmacology. 2005;61(12):921-8.  Salvi F, Morichi V, Grilli A, Lancioni L, Spazzafumo L, Polonara S, Abbatecola AM, De Tommaso G, Dessi-Fulgheri P, Lattanzio F. Screening for frailty in elderly emergency department patients by using the Identification of Seniors At Risk (ISAR). The journal of nutrition, health & aging. 2012;16(4):313-8.  Sánchez E, Vidán MT, Serra JA, Fernández-Avilés F, Bueno H. Prevalence of geriatric syndromes and impact on clinical and functional outcomes in older patients with acute cardiac diseases. Heart. 2011;97(19):1602-6.  Sanchis J, Bonanad C, Ruiz V, Fernández J, García-Blas S, Mainar L, Ventura S, Rodríguez-Borja E, Chorro FJ, Hermenegildo C, Bertomeu-González V. Frailty and other geriatric conditions for risk stratification of older patients with acute coronary syndrome. American heart journal. 2014;168(5):784-91.  Schmader KE, Hanlon JT, Pieper CF, Sloane R, Ruby CM, Twersky J, Francis SD, Branch LG, Lindblad CI, Artz M, Weinberger M. Effects of geriatric evaluation and management on adverse drug reactions and suboptimal prescribing in the frail elderly. The American journal of medicine. 2004;116(6):394-401.  Schultz M, Rosted E, Sanders S. Frailty is associated with a history with more falls in elderly hospitalised patients. Dan Med J. 2015;62(6).  Singh M, Rihal CS, Lennon RJ, Spertus JA, Nair KS, Roger VL. Influence of frailty and health status on outcomes in patients with coronary disease undergoing percutaneous revascularization. Circulation: Cardiovascular Quality and Outcomes. 2011;4(5):496-502.  Söderback I. Hospital discharge among frail elderly people: a pilot study in Sweden. Occupational therapy international. 2008;15(1):18-31.  Stiffler KA, Finley A, Midha S, Wilber ST. Frailty assessment in the emergency department. The Journal of emergency medicine. 2013;45(2):291-8.  Stretton CM, Latham NK, Carter KN, Lee AC, Anderson CS. Determinants of physical health in frail older people: the importance of self-efficacy. Clinical rehabilitation. 2006;20(4):357-66.  Studenski S, Hayes RP, Leibowitz RQ, Bode R, Lavery L, Walston J, Duncan P, Perera S. Clinical Global Impression of Change in Physical Frailty: development of a measure based on clinical judgment. Journal of the American Geriatrics Society. 2004;52(9):1560-6.  Subbe CP, Burford C, Le Jeune I, Masterton-Smith C, Ward D. Relationship between input and output in acute medicine–secondary analysis of the Society for Acute Medicine's benchmarking audit 2013 (SAMBA ‘13). Clinical Medicine. 2015;15(1):15-9.  Sujino Y, Tanno J, Nakano S, Funada S, Hosoi Y, Senbonmatsu T, Nishimura S. Impact of hypoalbuminemia, frailty, and body mass index on early prognosis in older patients (≥ 85 years) with ST-elevation myocardial infarction. Journal of cardiology. 2015;66(3):263-8.  Thomas C, Hestermann U, Walther S, Pfueller U, Hack M, Oster P, Mundt C, Weisbrod M. Prolonged activation EEG differentiates dementia with and without delirium in frail elderly. Journal of Neurology, Neurosurgery & Psychiatry. 2008;79(2):119-25.  Torres OH, Muñoz J, Ruiz D, Ris J, Gich I, Coma E, Gurguí M, Vázquez G. Outcome predictors of pneumonia in elderly patients: importance of functional assessment. Journal of the American Geriatrics Society. 2004;52(10):1603-9.  Uchmanowicz I, Lisiak M, Wontor R, Łoboz-Grudzień K. Frailty in patients with acute coronary syndrome: comparison between tools for comprehensive geriatric assessment and the Tilburg Frailty Indicator. Clinical interventions in aging. 2015;10:521.  Uchmanowicz I, Wleklik M, Gobbens RJ. Frailty syndrome and self-care ability in elderly patients with heart failure. Clinical interventions in aging. 2015;10:871.  van Iersel MB, Munneke M, Esselink RA, Benraad CE, Rikkert MG. Gait velocity and the Timed-Up-and-Go test were sensitive to changes in mobility in frail elderly patients. Journal of clinical epidemiology. 2008;61(2):186-91.  Velanovich V, Antoine H, Swartz A, Peters D, Rubinfeld I. Accumulating deficits model of frailty and postoperative mortality and morbidity: its application to a national database. journal of surgical research. 2013;183(1):104-10.  Vicente V, Ekebergh M, Castren M, Sjöstrand F, Svensson L, Sundström BW. Differentiating frailty in older people using the Swedish ambulance service: a retrospective audit. International emergency nursing. 2012;20(4):228-35.  Vidán MT, Sánchez E, Fernández‐Avilés F, Serra‐Rexach JA, Ortiz J, Bueno H. FRAIL‐HF, a Study to Evaluate the Clinical Complexity of Heart Failure in Nondependent Older Patients: Rationale, Methods and Baseline Characteristics. Clinical cardiology. 2014;37(12):725-32.  Villanyi D, Fok M, Wong RY. Medication reconciliation: identifying medication discrepancies in acutely ill hospitalized older adults. The American journal of geriatric pharmacotherapy. 2011;9(5):339-44.  Vitagliano G, Curtis JP, Concato J, Feinstein AR, Radford MJ, Krumholz HM. Association Between Functional Status and Use and Effectiveness of Beta‐Blocker Prophylaxis in Elderly Survivors of Acute Myocardial Infarction. Journal of the American Geriatrics Society. 2004;52(4):495-501.  Vivanti AP, McDonald CK, Palmer MA, Sinnott M. Malnutrition associated with increased risk of frail mechanical falls among older people presenting to an emergency department. Emergency Medicine Australasia. 2009;21(5):386-94.  Wallis SJ, Wall J, Biram RW, Romero-Ortuno R. Association of the clinical frailty scale with hospital outcomes. QJM: An International Journal of Medicine. 2015;108(12):943-9.  White HD, Westerhout CM, Alexander KP, Roe MT, Winters KJ, Cyr DD, Fox KA, Prabhakaran D, Hochman JS, Armstrong PW, Ohman EM. Frailty is associated with worse outcomes in non-ST-segment elevation acute coronary syndromes: Insights from the TaRgeted platelet Inhibition to cLarify the Optimal strateGy to medicallY manage Acute Coronary Syndromes (TRILOGY ACS) trial. European Heart Journal: Acute Cardiovascular Care. 2016;5(3):231-42.  Wilhelmson K, Duner A, Eklund K, Gosman-Hedström G, Blomberg S, Hasson H, Gustafsson H, Landahl S, Dahlin-Ivanoff S. Design of a randomized controlled study of a multi-professional and multidimensional intervention targeting frail elderly people. BMC geriatrics. 2011;11(1):24.  Wong RY, Miller WC. Adverse outcomes following hospitalization in acutely ill older patients. BMC geriatrics. 2008;8(1):10.  Wright RM, Sloane R, Pieper CF, Ruby-Scelsi C, Twersky J, Schmader KE, Hanlon JT. Underuse of indicated medications among physically frail older US veterans at the time of hospital discharge: results of a cross-sectional analysis of data from the Geriatric Evaluation and Management Drug Study. The American journal of geriatric pharmacotherapy. 2009;7(5):271-80.  Young J, Robinson M, Chell S, Sanderson D, Chaplin S, Burns E, Fear J. A prospective baseline study of frail older people before the introduction of an intermediate care service. Health & social care in the community. 2005;13(4):307-12.  Zorman JV, Lusa L, Strle F, Maraspin V. Bacterial infection in elderly nursing home and community-based patients: a prospective cohort study. Infection. 2013;41(5):909-16. |

Additional file 1: **Table S3 Descriptive characteristics of the articles which did not include a frailty measure**

|  | All | Geriatrics | Emergency Department | General Medicine | Cardiology | Orthopedics | Intensive Care Unit | Oncology | Surgery | Prehospital | General Medicine & Surgery | General Medicine & Geriatrics |
| --- | --- | --- | --- | --- | --- | --- | --- | --- | --- | --- | --- | --- |
| **Articles n (%)** | 413 | 112 | 52 | 43 | 20 | 47 | 6 | 1 | 4 | 11 | 7 | 8 |
| **Year n (%)** |  |  |  |  |  |  |  |  |  |  |  |  |
| 2011-2015 | 177 (42.9) | 42 (37.5) | 27 (51.9) | 20 (46.5) | 6 (30.0) | 16 (34.0) | 4 (66.7) | 0 | 4 (100.0) | 7 (63.6) | 5 (71.4) | 5 (62.5) |
| 2006-2010 | 139 (33.7) | 43 (38.4) | 11 (21.2) | 13 (30.2) | 8 (40.0) | 18 (38.3) | 1 (16.7) | 0 | 0 | 2 (18.2) |  | 3 (37.5) |
| 2000-2005 | 97 (23.5) | 27 (24.1) | 14 (26.9) | 10 (23.3) | 6 (30.0) | 13 (27.7) | 1 (16.7) | 1 (100.0) | 0 | 2 (18.2) | 2 (28.6) | 0 |
| **Language n (%)** | | |  |  |  |  |  |  |  |  |  |  |
| English | 383 (92.7) | 99 (88.4) | 50 (96.2) | 42 (97.7) | 20 (100.0) | 44 (93.6) | 3 (50.0) | 1 (100.0) | 4 (100.0) | 11 (100.0) | 7 (100.0) | 8 (100.0) |
| French | 11 (2.7) | 6 (5.4) | 1 (1.9) | 0 | 0 | 1 (2.1) | 0 | 0 | 0 | 0 | 0 | 0 |
| Spanish | 7 (1.7) | 3 (2.7) | 0 | 1 (2.3) | 0 | 1 (2.1) | 1 (16.7) | 0 | 0 | 0 | 0 | 0 |
| Italian | 5 (1.2) | 0 | 1 (1.9) | 0 | 0 | 1 (2.1) | 1 (16.7) | 0 | 0 | 0 | 0 | 0 |
| Dutch | 4 (1.0) | 1 (.9) | 0 | 0 | 0 | 0 | 1 (16.7) | 0 | 0 | 0 | 0 | 0 |
| German | 2 (.5) | 2 (1.8) | 0 | 0 | 0 | 0 | 0 | 0 | 0 | 0 | 0 | 0 |
| Portuguese | 1 (.2) | 1 (.9) | 0 | 0 | 0 | 0 | 0 | 0 | 0 | 0 | 0 | 0 |
| **Country n (%)** | | | | |  |  |  |  |  |  |  |  |
| United Kingdom | 77 (18.6) | 18 (16.1) | 11 (21.2) | 6 (14.0) | 2 (10.0) | 14 (29.8) | 0 | 1 (100.0) | 2 (50.0) | 4 (36.4) | 0 | 1 (12.5) |
| United States | 67 (16.2) | 9 (8.0) | 9 (17.3) | 6 (14.0) | 7 (35.0) | 5 (10.6) | 1 (16.7) | 0 | 2 (50.0) | 3 (27.3) | 3 (42.9) | 0 |
| France | 48 (11.6) | 25 (22.3) | 4 (7.7) | 5 (11.6) | 2 (10.0) | 1 (2.1) | 0 | 0 | 0 | 0 | 0 | 2 (25.0) |
| Italy | 38 (9.2) | 10 (8.9) | 10 (19.2) | 9 (20.9) | 0 | 2 (4.3) | 1 (16.7) | 0 | 0 | 0 | 0 | 2 (25.0) |
| Australia/New Zealand | 32 (7.7) | 3 (2.7) | 3 (5.8) | 4 (9.3) | 2 (10.0) | 5 (10.6) | 1 (16.7) | 0 | 0 | 0 | 0 | 0 |
| Canada | 17 (4.1) | 4 (3.6) | 3 (5.8) | 0 | 2 (10.0) | 4 (8.5) | 0 | 0 | 0 | 1 (9.1) | 1 (14.3) | 0 |
| Netherlands | 14 (3.4) | 5 (4.5) | 0 | 0 | 1 (5.0) | 1 (2.1) | 1 (16.7) | 0 | 0 | 0 | 2 (28.6) | 0 |
| Spain | 14 (3.4) | 6 (5.4) | 3 (5.8) | 1 (2.3) | 0 | 1 (2.1) | 1 (16.7) | 0 | 0 | 0 | 0 | 0 |
| Sweden | 13 (3.1) | 1 (0.9) | 2 (3.8) | 1 (2.3) | 0 | 4 (8.5) | 0 | 0 | 0 | 0 | 1 (14.3) | 0 |
| Finland | 12 (2.9) | 8 (7.1) | 0 | 3 (7.0) | 0 | 0 | 0 | 0 | 0 | 0 | 0 | 0 |
| Norway | 11 (2.7) | 0 | 0 | 0 | 1 (5.0) | 6 (12.2) | 0 | 0 | 0 | 0 | 0 | 0 |
| Israel | 10 (2.4) | 5 (4.5) | 1 (1.9) | 2 (4.7) | 0 | 1 (2.1) | 0 | 0 | 0 | 0 | 0 | 0 |
| Other Europe^1^ | 36 (8.7) | 14 (12.5) | 4 (7.7) | 5 (11.6) | 2 (10.0) | 1 (2.1) | 1 (16.7) | 0 | 0 | 1 (9.1) | 0 | 2 (25.0) |
| Other^2^ | 24 (5.8) | 4 (3.6) | 2 (3.8) | 1 (2.3) | 1 (5.0) | 2 (4.3) | 0 | 0 | 0 | 2 (18.2) | 0 | 1 (12.5) |

We only stratified by disciplines that have been included in at least 2 articles that measured frailty

^1^Austria; Belgium; Denmark; Germany; Iceland and Finland; Ireland; Portugal; Switzerland; Turkey; Multiple European countries

^2^Australia and Scotland; Brazil; China; Japan; Nigeria, Sudan, Tanzania, and UK; Singapore; Taiwan; USA and Canada; Multiple countries

B

A

^1^

^1^

Additional file 1 :**Figure S1. Proportion of articles across disciplines. A. Articles which included a frailty measure B. Articles which did not include a frailty measure**

^1^Combined units in which only one study was conducted

A

B

Additional file 1: **Figure S2 Proportion of articles by year of publication A. Articles which included a frailty measure B. Articles which did not include a frailty measure**

We only stratified by disciplines that have been included in at least 2 articles that measured frailty

^1^

Additional file 1:  **Figure S3 Proportion of articles based on who assessed frailty**

We only stratified by disciplines that have been included in at least 2 articles that measured frailty

^1^Next of kin or nurse in charge; well trained personnel; part of the assessment was done by researcher and another part by geriatrician

^1^

^2^

Additional file 1: **Figure S4 Proportion of articles based on type of frailty measure used and by year of publication**

^1^Tools that were not developed specifically for measuring frailty

^2^Operationalized definitions of frailty (for example, ≥65 years of age plus 3 comorbidities)

^1^

Additional file 1: **Figure S5 Number of articles for each most commonly used frailty measure**

^1^The Geriatric Evaluation and Management (GEM) drug study criterion was meeting at least 2 of the following 10 criteria: dependence in at least one activity of daily living, stroke within 3 months, previous falls, difficulty ambulating, malnutrition, dementia, depression, unplanned admission in the last 3 months, prolonged bed rest, or incontinence
